# Patients With Myeloproliferative Neoplasms Harbor High Frequencies of CD8 T Cell-Platelet Aggregates Associated With T Cell Suppression

**DOI:** 10.3389/fimmu.2022.866610

**Published:** 2022-05-06

**Authors:** Ana Micaela Carnaz Simões, Morten Orebo Holmström, Pia Aehnlich, Anne Rahbech, Marlies J. W. Peeters, Aneta Radziwon-Balicka, Carlos Zamora, Tobias Wirenfeldt Klausen, Vibe Skov, Lasse Kjær, Christina Ellervik, Daniel El Fassi, Silvia Vidal, Hans Carl Hasselbalch, Mads Hald Andersen, Per thor Straten

**Affiliations:** ^1^ Department of Oncology, National Center for Cancer Immune Therapy (CCIT-DK), Herlev University Hospital, Herlev, Denmark; ^2^ IIB-Sant Pau- Institut Rec. Hospital de la Santa Creu i Sant Pau, Barcelona, Spain; ^3^ Department of Hematology, Zealand University Hospital, Roskilde, Denmark; ^4^ Department of Clinical Medicine, Faculty of Health and Medical Sciences, University of Copenhagen, Copenhagen, Denmark; ^5^ Department of Laboratory Medicine, Boston Children’s Hospital, Harvard Medical School, Boston, MA, United States; ^6^ Department of Data and Innovation Support, Region Zealand, Sorø, Denmark; ^7^ Department of Hematology, Rigshospitalet University Hospital, Copenhagen, Denmark; ^8^ Department of Immunology and Microbiology, Faculty of Health and Medical Sciences, University of Copenhagen, Copenhagen, Denmark

**Keywords:** platelets, platelet-bound T cells, platelet-T cell aggregates, Myeloproliferative Neoplasms (MPN), CALR mutation, JAK2 mutation

## Abstract

Myeloproliferative neoplasms (MPN) are chronic cancers of the hematopoietic stem cells in the bone marrow, and patients often harbor elevated numbers of circulating platelets (PLT). We investigated the frequencies of circulating PLT-lymphocyte aggregates in MPN patients and the effect of PLT-binding on CD8 T cell function. The phenotype of these aggregates was evaluated in 50 MPN patients and 24 controls, using flow cytometry. *In vitro* studies compared the proliferation, cytokine release, and cytoxicity of PLT-bound and PLT-free CD8 T cells. Frequencies of PLT-CD8 T cell aggregates, were significantly elevated in MPN patients. Advanced disease stage and *CALR* mutation associated with the highest aggregate frequencies with a predominance of PLT-binding to antigen-experienced CD8 T cells. PLT-bound CD8 T cells showed reduction in proliferation and cytotoxic capacity. Our data suggest that CD8 T cell responses are jeopardized in MPN patients. *JAK2* and *CALR* exon 9 mutations – the two predominant driver mutations in MPN – are targets for natural T cell responses in MPN patients. Moreover, MPN patients have more infections compared to background. Thus, PLT binding to antigen experienced CD8 T cells could play a role in the inadequacy of the immune system to control MPN disease progression and prevent recurrent infections.

## Introduction

The Philadelphia chromosome-negative myeloproliferative neoplasms (MPN) comprise a heterogeneous group of diseases characterized by the clonal expansion of transformed hematopoietic stem cells (1, 2). In the early stages of MPN – essential thrombocythemia (ET) and polycythemia vera (PV) – platelet counts are variably elevated. The advanced MPN stage, primary myelofibrosis (PMF), is characterized by progressive bone marrow fibrosis and development of cytopenia (1). The overproduction of peripheral blood cells occurs primarily due to mutations in the *janus kinase 2* (*JAK2*), *calreticulin* (*CALR*), or *myeloproliferative leukemia* (*MPL*) genes, leading to the constitutive activation of the JAK-STAT pathway [reviewed by Vainchenker et al. (2)]. However, up to 15% of patients do not harbor any driver mutation – known as triple-negative MPN (3).

*JAK2* and *CALR* mutations generate cancer-specific neoantigens that are recognized by effector T cells (4, 5). Previous studies generated *JAK2* ^V617F^-specific CD8 T cell cultures from a healthy donor, which recognized and selectively killed *JAK2*-mutated cancer cells (4). In *CALR*-mutant MPN patients, we observed spontaneous and frequent immune responses against epitopes derived from the mutant *CALR* terminus, mediated by CD4^+^ T cells (5). Despite recognizing and killing autologous *CALR*-mutant cells (6), T cells derived from PMF patients elicited significantly reduced responses compared to ET-derived T cells (5). Remarkably, stronger and more frequent responses against CALR neoepitope were observed in healthy individuals (7) as well as in asymptomatic individuals harboring a low *CALR*-mutant allelic burden (8). These results support the hypothesis that *CALR*-mutant MPN evolves due to loss of immune-mediated tumor control.

Mounting evidence points towards a severe immune dysregulation in patients with MPN (8, 9), with both early- and advanced-stage patients exhibiting increased levels of several inflammatory cytokines (10–12) and a dysregulation in the frequency of circulating immune cells (13–16). Data on interferon-alpha (IFNα) efficacy in MPN patients further supports the theory of immune suppression: IFNα is an immunostimulatory drug capable of inducing long-lasting hematological and molecular remission in these patients (17, 18). As IFNα treatment results in marked alterations in the immune phenotype of patients (15, 19, 20) it is speculated that one of its mechanisms of action is the ability to induce an immune response against the malignant cells. Taken together, these data suggest that deregulation of the immune system is an important pathogenic factor for the development and evolvement of MPN.

PLT have been extensively described as crucial players in cancer development, progression, and metastasis (21, 22). Activated PLT (act-PLT) can release a wide range of molecules that promote tumor cell proliferation and maintenance of tumor integrity. Recently, Rachidi et al. have shown that transforming growth factor-beta (TGFβ) and lactate, released from PLT, can inhibit T cell function and promote resistance to adoptive T cell therapy in murine models (23). Increased circulating PLT-T cell aggregates were reported in lung cancer patients compared to healthy controls (24). Moreover, *in vitro* studies showed that these PLT-bound T cell aggregates exhibited a reduced proliferative capacity and released lower levels of proinflammatory cytokines than PLT-free T cells (25–27). Interestingly, expression of CD62P, a marker for platelet activation, is increased in MPN patients (28). PLT-monocyte and PLT-neutrophil aggregates have been previously studied in MPN (28, 29), but, to our knowledge, the presence of PLT-T cell aggregates in MPN patients has not been investigated.

As PLT levels are generally elevated in patients with MPN, and all three driver mutations affect the megakaryocytes, it has been speculated that PLT could interact with immune and tumor cells, thus facilitating tumor immune escape in MPN (30). In the present study, we evaluated the frequency of circulating PLT-lymphocyte aggregates in MPN and found that these patients display markedly higher PLT-T cell aggregates than healthy controls. Furthermore, our *in vitro* studies suggest that the binding of PLT decrease T cell functionality. Hence, we hypothesize that dampened T cell responses potentially jeopardize reactivity to transformed cells as well as preventive responses to infections.

## Materials and Methods

### Patient Population

Fifty patients diagnosed with MPN [according to the 2016 WHO classification (31)] were included in this study. Four asymptomatic individuals harboring a low *CALR*-mutant allelic burden with clonal hematopoiesis of indeterminate potential (*CALR*-mutant CHIP), from the GESUS cohort (32), were also included. The detailed baseline clinical parameters from the patient population are summarized in **Table 1**. For comparison purposes, 24 age-matched healthy controls (HC) with a median age of 57 years were included. The study was approved by the local ethics committee at Zealand Region (SJ-175, SJ-452, SJ-456 and SJ-585) and conducted according to the provisions of the Declaration of Helsinki. Written informed consent was obtained from all patients and healthy volunteers prior to the beginning of the study.

**Table 1:** Baseline Characteristics of the MPN study population.

Characteristics	MPN Patients (n = SO)
Median Age [range]	
at Diagnosis	58 [33-79]
at Sample	67 [42-81]
Median Time from Diagnosis [range]	7[0-24]
Gender, n (%)	
Female/male	30 (60%) / 20 (40%)
Median PLTCount (PLTx 109/L) [range]	325 [105-809]
Driver Mutation, n (%)	
CALR	29 (58%)
JAK2	16 (32%)
MPL	2 (4%)
Triple Negative	3 (6%)
Diagnosis, n (%)	
Healthy CALR mutation	4 (8%)
ET	11 (22%)
PV	11 (22%)
PreMF	7 (14%)
PMF	17 (34%)
ATI, n (%)	
None	9 (18%)
Aspirin	34 (68%)
Clopidogrel	3 (6%)
Apixabane	1 (2%)
Aspirin + Clopidogrel	2 (4%)
Aspirin + Dipyridamole	1 (2%)
CRT, n (%)	
None	11 (22%)
HU	11 (22%)
IFN-a	16 (32%)
ANA	6 (12%)
ANA+ HU	3 (6%)
Momelotinib	1 (2%)
Phlebotomy	2 (4%)
Hematological Response, n (%)	
CR/Non-CR	31 (62%) / 15 (30%)
Status, n (%)	
Alive/Deceased	44 (88%) / 6 (12%)

PV, Polycythemia Vera; ET, Essesntial Thrombocythemia; PMF, Primary Myelofibrosis; ATI, Anti-thrombotic Therapy; CRT, Cell Reduction Therapy; IFN, Interferon; HU, Hydroxyurea; ANA, Anagrelide.

### Isolation of Mononuclear Cells From Peripheral Blood and Bone Marrow Samples

Peripheral blood was collected from MPN patients, age-matched HC and young healthy volunteers. The last group was used for the *in vitro* studies. Bone marrow aspirates were collected from seven *JAK2-*mutated MPN patients (ET = 1; PV = 5; PMF = 1).

Peripheral blood mononuclear cells (PBMC) and bone marrow mononuclear cells (BMNC) were isolated from venous blood and bone marrow aspirate, respectively, by density gradient as described elsewhere (33). The cells were then used immediately or cryopreserved.

### Phenotyping PLT-Bound Immune Cells

Frequencies of PLT-binding to lymphocytes, as well as the activation status of bound PLT were detected using flow cytometry, an established technique to detect these aggregates (25–27). Platelet-bound T cells were evaluated in fresh and cryopreserved PMBC from MPN patients, and age-matched HC (Panel I, **Supplemental Table 1**). A similar setup was used to compare PLT-binding in isolated PBMC and BMNC from MPN (Panel II, **Supplemental Table 1**).

E*x vivo* PLT-binding to antigen-specific T cells in MPN and HC was evaluated in 10 x 10^6^ PBMC stained with CMV- and FLU-loaded MHC-multimers and analyzed by flow cytometry (Panel III, **Supplemental Table 1**). See the **Supplemental Material** for the detailed methodology.

### Isolation of Platelets

Blood was collected from young healthy volunteers who had not taken any drugs known to affect PLT function, for at least 14 days prior to the study. Venous peripheral blood was collected into acid citrate dextrose solution A tubes (Greiner Bio-One) and the PLT isolation procedure was adapted from Radomski et al. (34). Detailed methodology is available in the **Supplemental Material**.

### PLT-Binding to Stimulated CD8 T Cells

CD8 T cells from young healthy volunteers were stimulated with different concentrations of an anti-OKT-3 antibody or CMV-peptide. For OKT-3 stimulation, CD8 T cells were first isolated from PBMC using the MagniSort™ Human CD8 T cell Enrichment Kit (Thermofisher) following the manufacturer instructions. The cells were rested overnight, at 37°C and 5% CO_2_, and stimulated for three days with high (500 ng/ml), intermediate (20 ng/ml) or low (0.5 ng/ml) concentrations of plate-coated anti-CD3 (clone: OKT-3, Thermofisher), at a cell density of 1 x 10^6^ cells/ml. For CMV-peptide activation 4 - 5 x 10^6^ PBMC/ml from HLA-A2 positive healthy volunteers were stimulated with 20 nM CMV peptide and 120 U/ml IL-2 for seven days, followed by an additional overnight restimulation. After the activation period, the stimulated cells were co-cultured with allogenic PLT and PLT-binding was analyzed by flow cytometry (OKT-3: Panel IV; CMV-MHC-multimer staining: Panel V; **Supplemental Table 1**).

### Lymphocyte and PLT Co-Culture

OKT-3 stimulated CD8 T cells, CMV-stimulated PBMC and gp100-transduced (gp100^+^) T cells (see transduction protocol in the **Supplemental Material**) were co-cultured with allogenic PLT for one hour, at a PLT to lymphocyte ratio of 100: 1. The co-cultures were washed twice to remove any unbound PLT, before proceeding. To compare the sole effect of platelet-derived molecules on T cell functionality, gp100^+^ T cells were also co-cultured with PLT supernatant (sPLT), for one hour before removal by centrifugation.

### MHC-Multimer Staining

PLT-binding to antigen-specific T cells was evaluated by tetramer staining, in MPN patients and after PLT-lymphocyte co-culture. Empty loadable-MHC multimers (HLA-A*02:01; The Tetramer Shop) were loaded with HIV, CMV or FLU peptides, as described by Sanai et al. (35). See the supplemental material for the detailed protocol.

### *In Vitro* Characterization of PLT-Bound T Cell Function

PBMC from MPN patients were stimulated with OKT-3 for five hours or five days, to compare, respectively, the cytokine release and proliferation of PLT-bound and PLT-free T cells. Cytokine release and proliferation were evaluated using intracellular staining and CellTrace^®^ violet (CTV) (Thermofisher), respectively. Furthermore, gp100^+^ T cells were co-cultured with a melanoma cancer cell line (FM3) in the presence or absence of PLT, and real-time tumor cytotoxic capacity of T cells was evaluated with the xCELLigence system (Agilent, USA). Detailed methodology is available in the **Supplemental Material**.

### Statistical Analysis

Statistical analyses were performed using unpaired or paired T tests for comparisons of two groups, and unpaired non-parametric Kruskal-Wallis test for comparison of more than two groups. Single and multiple linear regression were used in the correlation studies, after applying a logarithmic transformation to the frequencies of PLT-bound lymphocytes. R^2^ and p values were calculated for each linear regression. All statistical tests were performed with a two-sided 95% confidence interval, at the 0.05 significant level, and the analyses were conducted using GraphPad software (version 8).

## Results

### MPN Patients Have Increased Frequencies of PLT-Bound CD8 T Cells Compared to HC

This study investigated the presence and frequencies of PLT-bound lymphocytes in MPN patients. The percentage of circulating PLT-bound lymphocytes in patients (N=50) and age-matched HC (N=24) was evaluated using flow cytometry. Since MPN patients display increased PLT activation, and act-PLT are thought to bind more avidly than resting PLT (36), we will focus on the act-PLT binding results.

**Figure 1:**
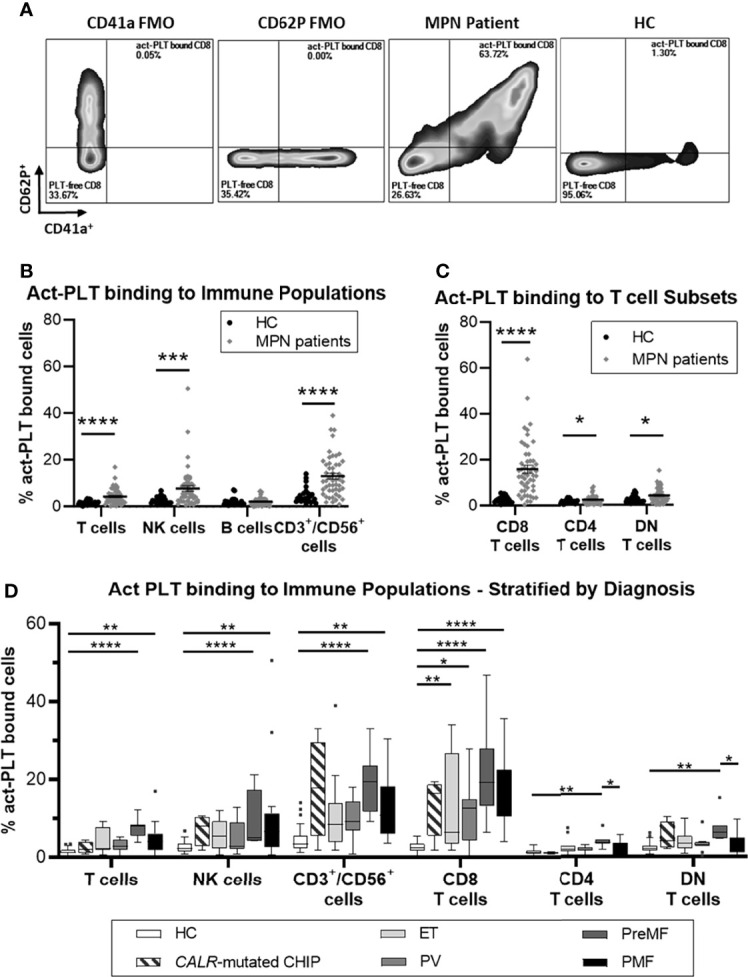
MPN patients have increased frequencies of circulating act-PLT bound cells compared to HC. Cryopreserved PBMCs from 50 patients with chronic myeloproliferative neoplasms (MPN) and 24 age-matched healthy controls (HC) were analyzed using flow cytometry (Panel I, **Supplemental Table 1**). Activated-Platelet (act-PLT) binding populations were identified by their concurrent expression of PLT markers (CD41a^+^/CD62P^+^) and immune specific markers, *i.e.*, CD3^+^/CD56^-^ (T cells), CD3^-^/CD19^+^ (B cells), CD3^-^/CD56^+^ (NK cells), CD3^+^/CD56^+^ (CD3^+^/CD56^+^ cells) CD3^+^/CD8^+^ (CD8 T cells), CD3^+^/CD4^+^ (CD4 T cells) and CD3^+^/CD8^-^/CD4^-^ (DN T cells). **(A)** Representative plots, comparing the frequency of act-PLT bound CD8 T cells in MPN and HC. CD41a and CD62P fluorescence minus one (FMO) were used as control. (See **Supplemental Figure 1** for detailed gating strategy). Frequencies of act-PLT binding to **(B)** the main lymphocytic populations and **(C)** the T cell subsets were compared in HC (•) and MPN patients (♦). **(D)** These frequencies were also compared across the different diagnoses within the patient population – essential thrombocythemia (ET; n = 11), Polycythemia Vera (PV; n = 11), Pre-myelofibrosis (PreMF; n = 7), and primary myelofibrosis (PMF; n = 17) - and including HC and asymptomatic individuals carrying a low *CALR*-mutant allelic burden (*CALR-*mutated CHIP; n = 4). All frequencies are shown as percentage of parent population. The horizontal lines and error whiskers represent the mean ± standard deviation of the mean, whereas boxplots follow the Tukey method. Unpaired T test was used to compare MPN patient and HC, whereas Kruskal-wallis test was used to compare the different mutation groups with n ≥ 6. Differences were considered significant when p < 0.05, as indicated with asterisks (*p < 0.05, **p < 0.01, ***p < 0.001, and ****p < 0.0001).

**Figure 1A** shows a representative plot comparing the frequency of act-PLT-CD8 T cells aggregates in MPN and HC (gating strategy in **Supplemental Figure 1**). Significantly higher frequencies of act-PLT-bound T cells, NK cells, and CD3^+^/CD56^+^ cells were observed in the patients compared to the HC (**Figure 1B**). Furthermore, MPN presented with significantly higher percentages of act-PLT bound CD8, CD4, and DN T cells than the control group (**Figure 1C**). Similar results were obtained for the frequencies of total-PLT (tPLT)-bound lymphocytes (**Supplemental Figure 2**). The results described above were obtained from cryopreserved samples. Therefore, the frequency of PLT-bound immune cells was also analyzed in freshly isolated and cryopreserved PBMC from MPN patients (**Supplemental Figure 3**). This comparison showed no significant differences between the samples. Comparison of the parent immune populations revealed no biologically relevant differences in the frequencies of live cells (mean_(MPN)_ = 99.9% vs mean_(HC)_ = 99.4%; **Supplemental Figure 4A**). The frequency of NK cells showed a decreasing trend (mean_(MPN)_ = 8% vs mean_(HC)_ = 14%; p = 0.095) in MPN patients, but no differences were observed in the frequencies of T cells, B cells, CD3^+^/CD56^+^ cells, or T cell subsets (**Supplemental Figure 4B**).

Analysis based on MPN diagnosis revealed that act-PLT-immune cell aggregates were elevated in advanced-stage patients (*i.e.*, PreMF and PMF) compared to HC (**Figure 1D**), particularly the PreMF patients. Although no statistics were performed due to the small sample size. the four *CALR*-mutated CHIP individuals had frequencies of act-PLT -CD8 T cells and -CD3^+^/CD56^+^ cells comparable to patients. The frequencies of act-PLT- (**Supplemental Figure 5A**) and tPLT- (**Supplemental Figure 5B**) bound lymphocytes were also compared across the different mutations: *CALR*-mutated patients exhibited increased frequencies of PLT-bound lymphocytes compared to HC and *JAK2*-mutated patients, although significance was not reached for the *CALR-JAK2* comparison. The two *MPL*-mutated patients presented with high levels in all PLT-bound populations, compared to other patient groups and HC. Lastly, triple-negative-MPN patients (n=3) exhibited frequencies PLT binding comparable to the HC population. However, a larger study population would be necessary to verify the results in MPL and triple-negative-MPN patients. Stratification according to cytoreductive therapy (CRT) revealed that patients receiving therapy other than IFNα (Not IFNα, n = 22) presented with the highest levels of act-PLT-lymphocyte aggregates (**Supplemental Figure 5C**).

Overall, these results point towards elevated frequencies of tPLT and act-PLT -bound lymphocytes, especially PLT -CD8 T cell, -CD3/CD56 cell and -NK cell aggregates, in MPN compared to the controls. Furthermore, *CALR*-mutated patients, patients receiving CRT other than IFNα, and patients with advanced disease tend to have the highest frequencies of these aggregates.

### PLT Count Correlates With the Frequencies of Circulating PLT-CD8 T Cell Aggregates in *JAK2* but Not *CALR* -Mutated MPN

To further explore potential clinical factors affecting PLT-binding in MPN patients, we investigated the association between these factors and the frequencies of act-PLT bound cells. To reduce the high variability seen for small subgroups, only *CALR-* or *JAK2-* mutated MPN were included.

**Figure 2:**
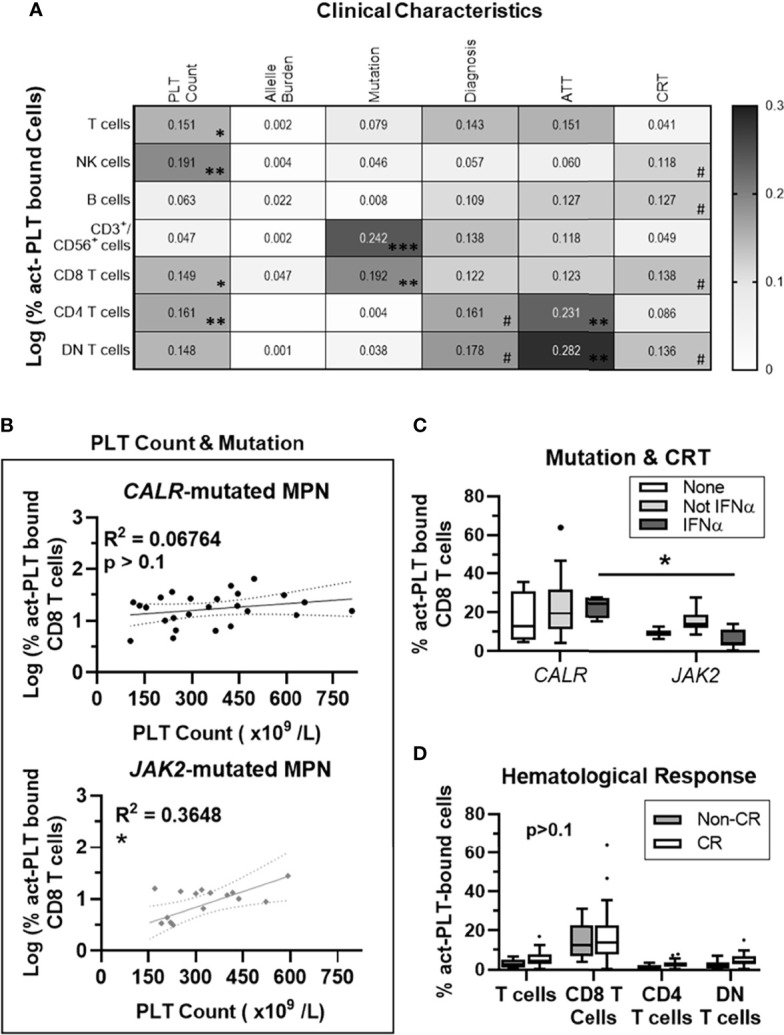
PLT Count correlates with act-PLT-binding to CD8 T cells in *JAK2*- but not *CALR*-mutated MPN patients. 41 MPN patients harboring either *CALR* or *JAK2* mutations were used for regression analysis. **(A)** The correlation between clinical characteristic (column factors) and the frequencies of activated-PLT (act-PLT) bound cells was analyzed, using single linear regressions. The goodness of fit (R^2^ value) for each linear regression model is shown as a heatmap (0 < R^2^ <0.3). The models were considered relevant when R^2^ > 0.10 and p-value < 0.05; **(B)** The correlation between PLT count and the frequency of act-PLT bound CD8 T cells was analyzed independently for *CALR*- and *JAK2*- mutated patients. Dotted lines represent the 95% confidence bands of the best-fit line. The frequencies of act-PLT-bound CD8 T cells were assessed after stratifying the patient population by **(C)** mutation (*CALR*: n = 25; *JAK2*: n = 16) and cytoreductive therapy (CRT) therapy. Patients either did not receive any CRT therapy (None, empty bars, n = 6), received IFNα therapy (IFNα, light-grey bars, n = 15), or received CRT other than IFNα (Not IFN α, dark-grey bars, n = 20). **(D)** Patients were stratified by hematological response – non-complete response (Non-CR; blue bars; n = 12) and complete response (CR; empty bars; n = 29). The frequencies of act-PLT bound T cells, CD8 T cells, CD4 T cells and DN T cells were compared within the stratified MPN population. All frequencies are shown as percentage of parent population, and boxplots follow the Tukey method. Kruskal-wallis test or multiple unpaired T test were used to compare the populations with n ≥ 6. Differences between groups were considered significant when p < 0.05. Asterisks represent *p < 0.05, **p < 0.01, ***p < 0.001, and ****p < 0.0001. # means the p value ranges from 0.05 < p < 0.1. PLT, Platelets; MPN, Chronic Myeloproliferative Neoplasms; AAT, Anti-thrombotic Therapy.

PLT count accounted for 19% of the variability in the frequency of act-PLT bound NK cells (R^2^ = 0.19; p = 0.004), while also correlating with act-PLT binding to T cells (R^2^ = 0.15; p = 0.012), CD8 T cells (R^2^ = 0.14; p = 0.013), CD4 T cells (R^2^ = 0.16; p = 0.009) and DN T cells (R^2^ = 0.14; p = 0.013) (**Figure 2A**). The driver mutation strongly correlated with a higher act-PLT binding to CD3^+^/CD56^+^ (R^2^ = 0.24; p=0.001) and CD8 T cells (R^2^ = 0.19; p = 0.004), with *JAK2*-mutated patients displaying lower frequencies of aggregates compared to *CALR*-mutant MPN (regression models in **Supplemental Table 2**). Finally, antithrombotic therapy (ATT), CRT and diagnosis showed some degree of correlation with act-PLT-binding to lymphocytes. However, the small sample sizes within each group prevents strong conclusions.

Multivariate linear regression analyses showed that PLT count and mutation type account for over 30% of the variability seen in the frequency of act-PLT-bound CD8 T cells (R^2^ = 0.30; p = 0.001). Interestingly, PLT count did not correlate with the frequency of act-PLT-CD8 T cell aggregates in patients with *CALR* mutation (R^2^ = 0.07; p = 0.209) but had a strong association in *JAK2*-mutated MPN (R^2^ = 0.37; p = 0.013) (**Figure 2B**). Separating patients by mutation and CRT showed that patients receiving IFNα revealed higher act-PLT-bound CD8 T cells in *CALR*- than *JAK2-* mutated patients (23% vs. 6%; p = 0.029) (**Figure 2C**). These results should be interpreted with caution due to the limited sample sizes in the IFNα-receiving *CALR*-mutated group (n = 4). Lastly, MPN patients with and without a complete hematological response showed similar levels of act-PLT immune cell aggregates (**Figure 2D**).

The regression analysis was also performed for the frequencies of tPLT-bound lymphocytes, which yielded similar results (**Supplemental Figures 6A–B**; Regression models in **Supplemental Table 2**). Interestingly, the mutant allele burden showed a significant association with tPLT-binding to CD8 T for *JAK2*- (R^2^ = 0.28; p = 0.033) but not *CALR-* (R^2^ = 0.02; p = 0.540) mutated patients (**Supplemental Figure 6C**). Although, this correlation did not stem from an association between the two characteristics (R^2^ = 0.03; p = 0.265; **Supplemental Figure 5D**), the allele burden was significantly higher in *CALR*- compared to *JAK2*- mutated MPN (36% vs 10%; p = 0.016; **Supplemental Figure 6E**).

Taken together, our results show that *JAK2* mutation strengthens the correlation between PLT count and the frequency of PLT-CD8 T cell aggregates. Additionally, *JAK2* mutations also increases the correlation between the mutant allele burden and tPLT-binding to CD8 T cells.

### Comparable Frequencies of PLT-Immune Cell Aggregates in the Peripheral Blood and Bone Marrow of MPN

**Figure 3:**
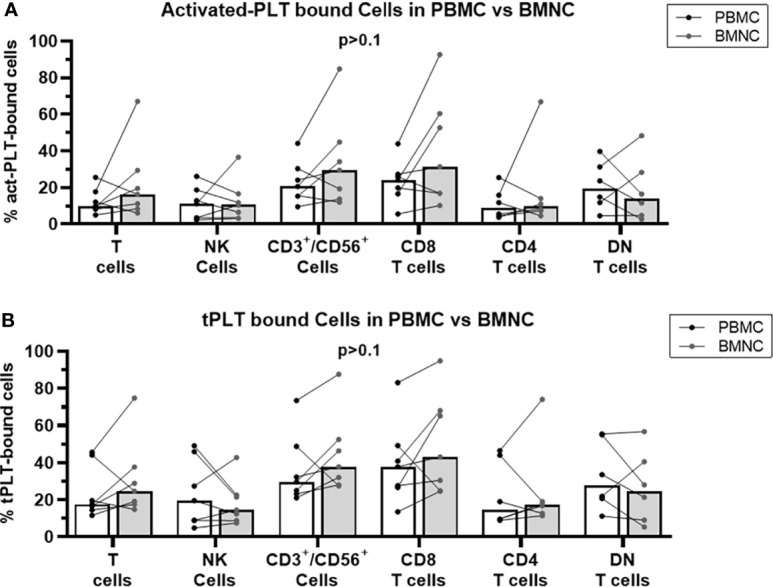
BMNC and PBMC show comparable frequencies of PLT-bound cells in MPN patients. PBMC (black dots and empty bars) and BMNC (grey dots and grey filled bars) were isolated from seven *JAK2-*mutated MPN patients (ET = 1; PV = 5; PMF = 1). **(A)** activated-PLT (act-PLT) bound lymphocytes and **(B)** total (tPLT)- bound lymphocytes were evaluated using flow cytometry (Panel II, **Supplemental Table 1**). The graph shows the frequencies of act-PLT-bound cells for different lymphocytic populations and all frequencies are shown as percentage of parent population. The bars represent the median frequency for each population. Multiple unpaired T tests were used to compared PLT bound cells in PBMC and BMNC samples, and differences were considered significant when p < 0.05.

Since the transformed cells in MPN reside in the bone marrow and PLT-bound T cells in the bone marrow may affect the local tumor-specific immune response, we analyzed the frequencies of PLT-lymphocyte aggregates in freshly isolated PBMC and BMNC from MPN patients. No significant differences in the frequencies of act-PLT-bound (**Figure 3A**) or tPLT-bound (**Figure 3B**) lymphocytes were observed between the two populations. Nevertheless, some patients displayed twice the frequencies of act-PLT-bound BMNC compared to act-PLT-bound PBMC. No differences were registered in the parent immune populations between the groups (data not shown). Therefore, our results suggest that peripheral blood and bone marrow of MPN patients display similar frequencies of PLT-immune cell aggregates.

### PLT Bind Preferentially to Antigen-Experienced CD8 T Cells

**Figure 4:**
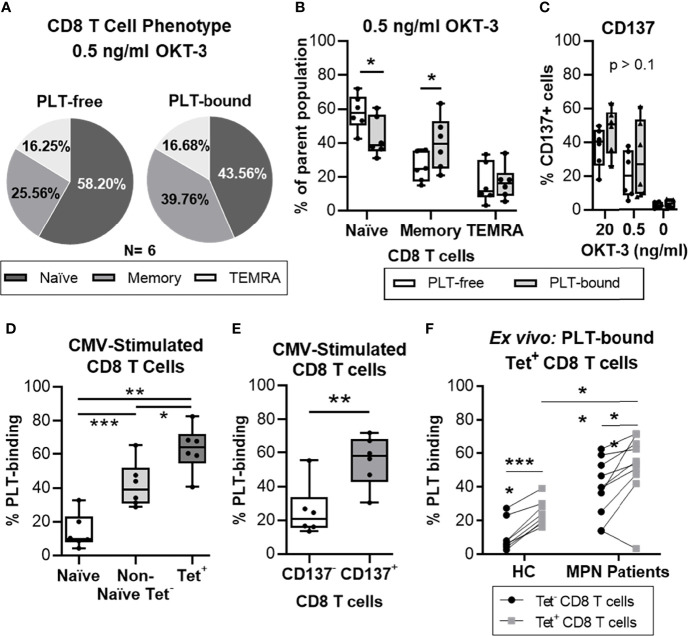
PLTs bind preferentially to antigen-specific CD8 T cells, *in vitro* and *ex vivo*. Isolated CD8 T cells from healthy donors were stimulated with OKT-3 (20 or 0.5 ng/ml) for three days. Afterwards, CD8 T cells were co-cultured with PLT for one hour (1:100 ratio), and PLT-binding was assessed by flow cytometry (Panel IV, **Supplemental Table 1**). **(A)** The frequency of Naïve (CCR7^+^/CD45RO^-^), Memory (CD45RO^+^) and terminally differentiated effector (TEMRA; CCR7^-^/CD45RO^-^) CD8 T cells is shown as the mean value of all donors (N = 6) in a pie chart. The percentages of **(B)** naïve, memory and TEMRA as well as **(C)** CD137^+^ cells are shown for each individual donor for PLT-bound (empty bars) and PLT-free (grey bars) CD8 T cells. Unpaired T test was used to compare the PLT-free and PLT-bound populations. Next, PBMC from healthy donors were stimulated twice with a CMV peptide. PBMC were co-cultured with PLT as described above and PLT-binding to CMV-specific CD8 T cells was evaluated using flow cytometry (Panel IV, **Supplemental Table 1**). Frequencies of PLT-binding to **(D)** Naïve (empty bar), Non-Naïve tetramer^-^ (Non-Naïve Tet^-^; CMV-MHC-tetramer^-^ and exclusion of CCR7^+^/CD45RO^-^; light grey bar) and Tetramer^+^ (Tet^+^; dark gray bar), and **(E)** non-activated (CD137^-^; empty bar) and activated (CD137^+^; grey bar) CD8 T cells were compared using paired non-parametric ANOVA analysis or T test, respectively (n = 6). **(F)** PBMC from age-matched healthy controls (HC; N = 8) and MPN patients (N = 10) were stained for Flu and CMV MHC-I tetramer, and the circulating frequencies of PLT-bound to MHC-tetramer negative (Tet^-^; •) and virus-specific (Tet^+^; ^▪^) CD8 T cells were analyzed by flow cytometry (Panel V). Paired T tests were used to compare unspecific and virus-specific frequencies, whereas unpaired T test was used to compare the virus-specific populations in HC and MPN patients. All frequencies are shown as percentage of parent population and the boxplots represent the median ± interquartile range of the populations, while the whiskers extend to the maximum and minimum values. Differences were considered significant when p<0.05, as indicated with asterisks (*p < 0.05, **p < 0.01, ***p < 0.001, and ****p < 0.0001).

To probe into the phenotype of PLT-bound T cells, PBMC from healthy volunteers were stimulated with different concentrations of OKT-3, co-cultured with allogenic PLT and analyzed by flow cytometry (N = 6, **Figure 4**). **Figure 4A** shows the mean distribution of the differentiation stages within the PLT-free and PLT-bound CD8 T cells, after low OKT-3 stimulation. PLT-bound CD8 T cells had a significantly higher frequency of memory cells (CD45RO^+^) than PLT-free CD8 T cells (26% vs 40%, p = 0.036) (**Figure 4B**), while PLT-free CD8 T cells harbored primarily a naïve (CCR7^+^/CD45RO^-^) phenotype (58% vs 44%, p = 0.042). Similar trends were observed in higher dose OKT-3, as well as in unstimulated cells (data not shown). No differences were observed in the frequencies of activated CD8 T cells (CD137^+^) within the PLT-free or PLT-bound populations (**Figure 4C**).

Next, we examined the PLT capacity to bind antigen-experienced CD8 T cells. PBMC from healthy volunteers were stimulated *in vitro* with a CMV peptide, co-cultured with PLT and analyzed by flow cytometry (N=6) (gating strategy in **Supplemental Figure 7**). We compared the frequencies of PLT-bound naïve, non-naïve cells not expressing MHC-tetramer (non-naïve tetramer^-^) and CMV-specific (tetramer^+^) CD8 T cells (**Figure 4D**). PLT-binding was highest in CMV-specific CD8 T cells (mean = 63%) compared to the non-naïve tetramer^-^ (mean = 42%; p = 0.026) and naïve (mean = 14%; p = 0.002) groups. Additionally, differences were observed between the naïve and the non-naïve tetramer^-^ populations (p = 0.0001), and a significant increase was found in PLT-binding to activated CD8 T cells compared to non-activated cells (mean of differences = 30%; p = 0.009; **Figure 4E**).

Lastly, circulating PLT-bound CMV- and influenza- specific CD8 T cells were evaluated in *ex vivo* unstimulated cells from MPN patients and age-matched HC (**Figure 4F**). The frequency of PLT-bound virus-specific CD8 T cells (tetramer^+^) was significantly increased compared to tetramer^-^ CD8 T cells, in MPN (mean of differences = 13%; p = 0.0096) and HC (mean of differences = 14%; p < 0.0001) groups. PLT-binding to virus-specific CD8 T cells was also significantly increased in MPN patients compared to the HC group (MPN = 52% vs. HC = 25%; p = 0.0018).

Taken together, these results show that PLT have a clear preference to bind antigen-experienced CD8 T cells. Importantly, similar analyses in MPN patients show this feature too.

### PLT-Binding Impacts the Proliferation and Killing Capacity of CD8 T Cells in MPN

**Figure 5:**
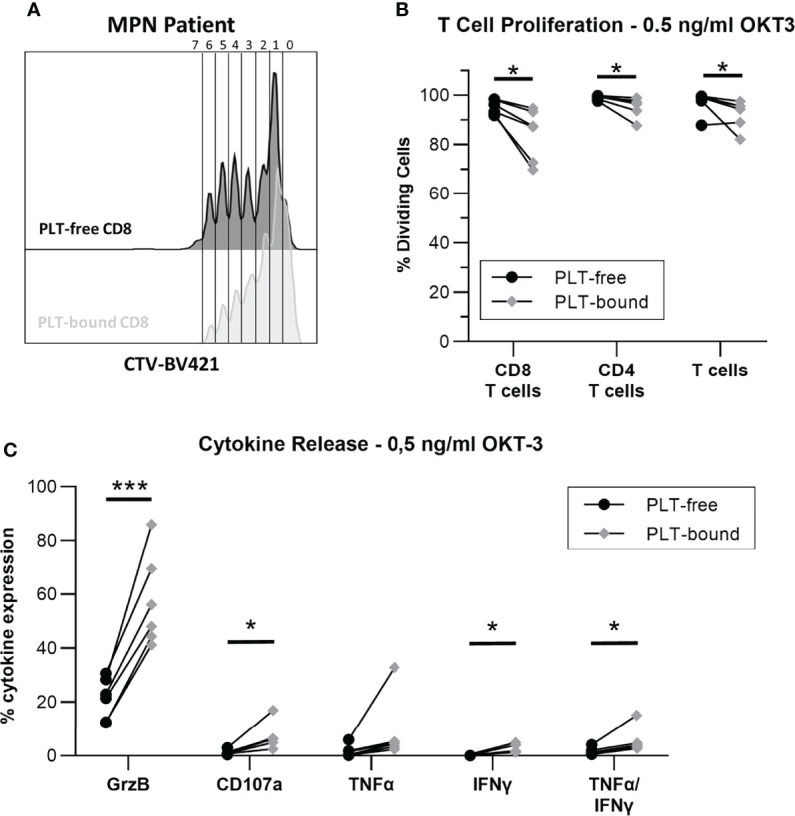
*In vitro* PLT-bound CD8 T cells from MPN patients show decreased proliferation compared to PLT-free CD8 T cells. Cryopreserved samples from six MPN were stimulated with OKT-3 (0.5ng/ml) for five hours (intracellular staining) or six days (Proliferation Assay). For the proliferation assay, PBMC were stained with CellTrace Violet^®^ (CTV) before OKT-3 stimulation. **(A)** A representative plot of the proliferation cycles in PLT-free (black) and PLT-bound (red) CD8 T cells is shown. **(B)** The frequency of proliferating PLT-free and PLT-bound CD8 T cells, CD4 T cells and total T cells are shown (n = 6) after five-day stimulation. **(C)** The release of interferon-gamma (IFN-γ), tumor necrosis factor-alpha (TNF-α) and Granzyme B (GrzB), as well as the expression of CD107a were compared in PLT-free and PLT-bound CD8 T cells (n = 6). All frequencies are shown as percentage of parent population. Paired T tests were used to compare PLT-free and PLT-bound frequencies and differences were considered significant when p < 0.05, as indicated with asterisks (*p < 0.05, **p < 0.01, ***p < 0.001, and ****p < 0.0001).

To investigate the impact of PLT-binding on CD8 T cell function, proliferation and cytokine release were evaluated in PBMC from MPN patients, after stimulation with OKT-3. **Figure 5A** shows a representative plot of PLT-bound and PLT-free CD8 T cell proliferation, after a 5-day OKT-3 stimulation. CD8 T cell proliferation is significantly reduced in PLT-bound compared to PLT-free cells in MPN patients (N=6, **Figure 5B**), with PLT-bound cells undergoing less divisions than PLT-free CD8 T cells. Conversely, after five-hour OKT-3 stimulation, the expression of IFNγ, TNFα and CD107a was increased in PLT-bound CD8 cells compared to the PLT-free population (**Figure 5C**).

**Figure 6:**
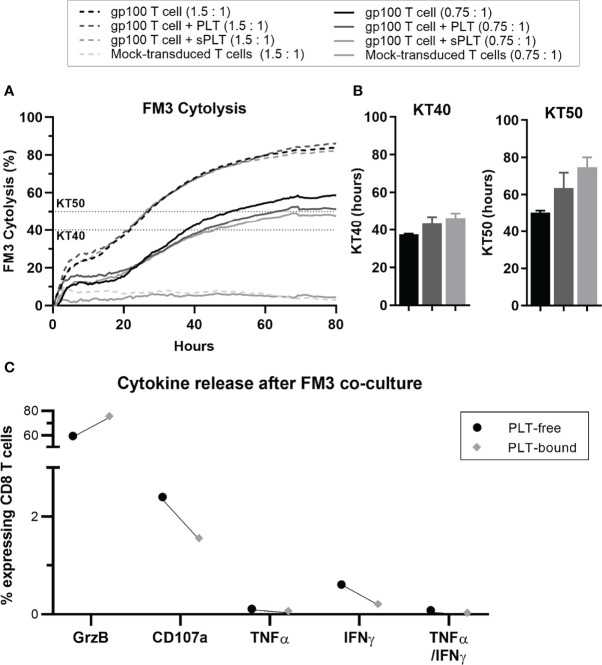
After prolonged PLT exposure, *in vitro* PLT-bound CD8 T cells show impaired killing capacity compared to PLT-free CD8 T cells. Gp100-transduced T cells were co-cultured with PLT (ratio 100:1) or PLT-supernatant (sPLT) for 1h, after which unbound PLT were washed off. **(A)** The xCELLigence system was used to evaluate FM3 cytolysis after co-culture with transduced T cells alone (GP100^+^T cells, green), transduced T cells + PLT (GP100^+^ T cells + PLT, red) or transduced T cells + PLT-supernatant (GP100^+^ T cells + sPLT, blue) at an effector to target ratio of 1.5: 1 (dotted lines) or 0.75:1 (full lines), for 80 hours (N = 1). **(B)** Killing time 40 (KT40) and 50 (KT50) (*i.e.*, hours until 40% or 50% FM3 cells were killed, respectively) was calculated for all conditions at a ratio of 0.75:1. **(C)** PLT were co-cultured with Gp100-transduced T cells for 48 hours, followed by a 48-hour co-culture with FM3 cell line. The cytokine release of interferon-gamma (IFN-γ), tumor necrosis factor-alpha (TNF-α) and Granzyme B (GrzB), as well as CD107a were compared in PLT-free and PLT-bound CD8 T cells (n = 1). Due to the small sample size statistical analyses were not performed (N = 1).

In a pilot study, we co-cultured gp100^+^-transduced T cells with PLT, before assessing the cytokine release and cytotoxicity of these aggregates against the gp100-expressing melanoma cell line FM3. Over an 80-hour period, CD8 T cells showed a reduction in cytolysis in the condition containing PLT compared to T cells alone (**Figure 6A**). Since this system cannot distinguish between PLT-bound and PLT-free within the same culture, a condition containing gp100^+^ T cells with PLT supernatant (sPLT) was added, which showed the highest cytolysis levels. Killing time 40 (KT40) and KT50 provided information on the number of hours necessary for effector cells to kill 40-50% of FM3. While KT50 for gp100^+^ T cells alone was 50.3 hours, the conditions containing PLT and sPLT registered 63.5 and 74.7 hours, respectively (**Figure 6B**). The cytokine release analysis after a 5-day co-culture with PLT and FM3 showed that PLT-bound CD8 T cells decreased the expression of IFN-γ and CD107a and increased Granzyme B, compared to PLT-free cells (**Figure 6C**).

These *in vitro* results point towards a decrease in the proliferation, cytokine release and cytotoxic capacity of CD8 T cells when exposed to prolonged/chronic PLT-binding.

## Discussion

Our results show that PLT count is the most relevant factor associated with PLT-binding. Additionally, we show an association between driver mutation and PLT-binding, with *JAK2*-mutants displaying lower levels of PLT-CD8 T cell aggregates than *CALR*-mutants. Interestingly, in *JAK2*-mutated MPN, the PLT count is highly correlated to the PLT binding, whereas no association is seen in *CALR*-mutants. These data are quite surprising, as both mutations confer aberrant activation of the thrombopoietin receptor leading to exacerbated JAK-STAT signaling (2). Hence, we expected that PLT binding would be independent of the driver mutation. As both the *JAK2-* and *CALR*- mutations have been identified in the lymphoid compartment (37, 38), the differences in PLT binding cannot be explained by different occurrences of driver mutations in the lymphoid cells. Instead, these data could indicate that even though the *CALR*-mutations activate the JAK-STAT pathway, it may also facilitate aberrant activation of other pathways, which are not triggered by the *JAK2^V617F^*-mutation. An increased mutant allele burden correlates with PLT-binding in *JAK2* but not *CALR* mutated patients, indicating that the PLT-T-cell binding mechanisms facilitated by the two mutations may be different. Lastly, we find it noteworthy that the triple-negative group shows low levels of PLT-binding, whereas MPN with *MPL* mutations – another thrombopoietin receptor mutation - exhibited high binding levels. These data further support our hypothesis that the increased PLT-T-cell interaction depends on aberrant activation of JAK-STAT signaling, as well as other pathways specific to the thrombopoietin receptor. Nevertheless, the small sample size in the triple-negative and the *MPL*-mutated populations prevents stronger conclusions.

Most MPN patients receive ATT and/or CRT drugs to reduce the peripheral blood counts and decrease the risk of thrombosis. Thus, we speculated that CRT could be correlated to decreased PLT-binding. However, this was only observed in patients with *JAK2*-mutated MPN for whom treatment with IFNα decreased the PLT-binding to CD8 T cells. This interesting finding supports the theory that IFNα enhances the tumor-specific immune response in patients (17, 18, 39). As MPN is an inflammatory disease and the PLT-binding could be mediated by the high levels of inflammation (36), it would be interesting to investigate PLT-binding in MPN patients treated with the clinically approved JAK1/2 inhibitor ruxolitinib, as this drug is a highly anti-inflammatory agent (40). Despite the encouraging results, the validation and understanding of our data require a bigger and more homogenous cohort.

As the bone marrow in MPN represents the actual tumor site, it was important to assess PLT-binding in BMNC. It may be challenging to estimate/compare the frequencies of PLT-immune cell aggregates in the peripheral blood and bone marrow, due to fibrosis and PB contamination. However, most of the patients included in the comparison of platelet-binding in the periphery and bone marrow are early stage PV (N=5) and ET (N=1) who do not present with fibrosis. Here we showed no difference in the frequency of aggregates in the peripheral blood and bone marrow samples. However, the small sample size could be masking a significant difference between the two groups, particularly for PLT-bound CD8 T cells and CD3^+^/CD56^+^ cells, where several patients display a higher frequency of aggregates at the tumor site compared to the peripheral blood.

Here we characterized the PLT-bound CD8 T cell aggregates *in vitro* after stimulation with OKT-3 or CMV-peptide. Our results strongly suggest a selective PLT-binding to memory and antigen-specific CD8 T cells. Moreover, our *in vitro* data showed a decrease in PLT-CD8 T cell proliferation in MPN samples, as well as a reduction in cytokine release and killing capacity of CD8 T cells in the presence of PLT. Previous studies have also shown that PLT-binding decreases T cell proliferation (25–27), but, to our knowledge, this is the first report on the effect of PLT-binding on T cell killing efficiency. Although cytotoxic CD8 T cells are often the primary mediators of anti-tumor specific cytolysis, we have previously shown low CALR-specific CD8 T cell-responses in MPN. Instead, these responses seem to be primarily mediated by CD4 T cells (5, 6). The high PLT-binding to CD8 T cells could explain these results. However, these hypotheses would require further investigation.

Interestingly, *CALR*-mutated CHIP individuals display a PLT-binding frequency close to that of MPN patients. This group was suggested to be in an immunoediting step of cancer elimination: they can mount strong CALR-specific responses but cannot eliminate all mutant cells (8). In that context, we suggest that PLT could be a factor preventing the full elimination of cancer cells by binding CD8 T cells and other lymphocytes, which could otherwise potentiate an effective anti-tumor immune response. Accordingly, we show high PLT-binding to NK cells in MPN patients compared to HC. NK cells have been shown to protect from metastasis formation, whereas PLT-binding to cancer cells allows these aggregates to evade NK cell-mediated killing (41–43). It is tempting to suggest that PLT can also bind NK cells, thus shielding the tumor cells from NK cell-mediated killing.

There is a strong association between MPN and autoimmune disorders, with one disease increasing the susceptibility to the other (30). Zamora et al. has shown that rheumatoid arthritis patients with a good prognosis have higher PLT-binding CD4 T cells than patients with worse prognosis and healthy controls (25), suggesting that PLT-binding helps prevent the flared immune reaction against self. Our *in vitro* data on proliferation and cytokine secretion upon PLT binding – showing less proliferation but more cytokine secretion – seem counterintuitive in this regard. However, Starossom et al. proposed a model for the PLT-binding to CD4 T cells in multiple sclerosis: during acute inflammation platelets can support CD4 T cells and promote their Th1/Th17 differentiation; once chronic inflammation sets in, exhausted platelets bind CD4 T cells and hamper the T cell function (27). This model helps explain our conflicting cytokine release data: when T cell were co-cultured with PLT for short periods, IFNγ and TNFα release increased; when PLT were co-cultured for over a 48-hour period, the cytokine release was decreased. Taken together, these data support the hypothesis that the high PLT-binding to T cells in chronically inflamed MPN may be impairing the CALR- and JAK2-specific immune responses, and thus allowing the disease to progress unrestrained.

The mechanism mediating the immune suppression by platelet-binding is not fully understood. TGF-β has been described as the main platelet-derived factor inhibiting *in vitro* T cell proliferation and cytokine release as well as *in vivo* tumor cell killing: Rachidi et al. have shown, in a murine model, an almost complete abrogation of tumor killing capacity by T cells in the presence of platelets supernatant (23). Therefore, one cannot exclude that the added effect of platelet-binding on T cell function may simply be the result of proximity. Nevertheless, Zamora et al. demonstrated that the platelet-binding immune suppression is mediated *via* P-selectin ligation to P-selecting glycoprotein ligand (PSGL)-1 on the surface of lymphocytes (26). Other studies have shown a negative effect of PSGL-1 signaling on T cell function (44), and more recently PSGL-1 was proposed as a new immune checkpoint (45). Studies on PSGL-1^-/-^ murine models have not only revealed an increase in proliferation, but also shown that P-selectin is the major receptor for PSGL-1 in activated T cells (44). This is in line with our *in vitro* and *ex vivo* results that platelet-binding occurs preferentially in memory and antigen-specific CD8 T cells. Future research evaluating anti-tumor T cell function, in the presence of a P-selectin/PSGL-1 blocking antibody would demonstrate the relevance of this interaction in tumor-specific immune responses. Lastly, a recent study has shown that platelets can upregulate their MHC-class I surface expression, which can downregulate CD8 T cell activity (46). However, gene expression profiling studies in MPN patients have shown a downregulation of human leucocyte antigen (HLA)-I, HLA-II, and HLA-related genes (47), making this mechanism less likely to occur in MPN.

In conclusion, we have shown that MPN patients have elevated levels of circulating PLT-bound lymphocytes, especially PLT- bound CD8 T and NK cells, compared to age-matched HC. Since advanced disease and the presence of *CALR* mutation associate with the highest frequency of these aggregates, we propose that the driver mutations may modulate PLT binding to lymphocytes differently. *In vitro* and *ex vivo* phenotype analysis of PLT-bound CD8 T cells show a predominant PLT-binding to antigen-experienced CD8 T cells. Further analysis suggests a lower proliferative and cytotoxic capacity of PLT-bound cells compared to PLT-free, as demonstrated by a decrease in PLT-CD8 T cell proliferation as well as a reduction in cytokine release and killing capacity of CD8 T cells in the presence of PLT. Finally, we demonstrate that PLT-binding occurs not only in circulation but also at the tumor site. Therefore, we propose that PLT bind antigen-experienced T cells in MPN and can potentially dampen their reactivity in future encounters with the antigen, thus increasing the risk of recurrent infections and promote tumor immune evasion. Further studies are required to understand the underlying mechanism.

## Data Statement Availability

The data generated in this study are available upon request from the corresponding author.

## Author Contributions

Contribution: AMCS, PA, AR, and AR-B performed research. AMCS, MOH, PA, VS, LK, CE, and DF collected data. AS performed the experimental design and data analysis. AMCS and TWK performed statistical analysis. AMCS, MOH, CZ, SV, HH, MHA and PS interpreted the data. AMCS and MOH wrote the manuscript, and PS revised it. SV and PS supervised the study. MOH, MHA and PS designed the study. All authors contributed to the article and approved the submitted version.

## Funding

This work was supported, in part, by Marie Skłodowska-Curie Actions – IMMUTRAIN (EU H2020 under the grant agreement number 641549) to PtS. Danish Council for Independent Research (grant no. DFF-1331-00095B), the Danish Cancer Society (grant no. R72-A4396-13-S2), The Danielsen Foundation, Axel Musfeldts fond, Dagmar Marshalls Fond, Else og Mogens Wedell- Wedellsborg Fond, AP Møller Fonden, and Den Bøhmske Fond.

## Conflict of Interest

The authors declare that the research was conducted in the absence of any commercial or financial relationships that could be construed as a potential conflict of interest.

## Publisher’s Note

All claims expressed in this article are solely those of the authors and do not necessarily represent those of their affiliated organizations, or those of the publisher, the editors and the reviewers. Any product that may be evaluated in this article, or claim that may be made by its manufacturer, is not guaranteed or endorsed by the publisher.
