# Corrigendum: Patients with myeloproliferative neoplasms harbor high frequencies of CD8 T cell-platelet aggregates associated with T cell suppression

**DOI:** 10.3389/fimmu.2022.970322

**Published:** 2022-08-05

**Authors:** Ana Micaela Carnaz Simões, Morten Orebo Holmström, Pia Aehnlich, Anne Rahbech, Marlies J. W. Peeters, Aneta Radziwon-Balicka, Carlos Zamora, Tobias Wirenfeldt Klausen, Vibe Skov, Lasse Kjær, Christina Ellervik, Daniel El Fassi, Silvia Vidal, Hans Carl Hasselbalch, Mads Hald Andersen, Per thor Straten

**Affiliations:** ^1^ Department of Oncology, National Center for Cancer Immune Therapy (CCIT-DK), Herlev University Hospital, Herlev, Denmark; ^2^ IIB-Sant Pau- Institut Rec. Hospital de la Santa Creu i Sant Pau, Barcelona, Spain; ^3^ Department of Hematology, Zealand University Hospital, Roskilde, Denmark; ^4^ Department of Clinical Medicine, Faculty of Health and Medical Sciences, University of Copenhagen, Copenhagen, Denmark; ^5^ Department of Laboratory Medicine, Boston Children’s Hospital, Harvard Medical School, Boston, MA, United States; ^6^ Department of Data and Innovation Support, Region Zealand, Sorø, Denmark; ^7^ Department of Hematology, Rigshospitalet University Hospital, Copenhagen, Denmark; ^8^ Department of Immunology and Microbiology, Faculty of Health and Medical Sciences, University of Copenhagen, Copenhagen, Denmark

**Keywords:** platelets, platelet-bound T cells, platelet-T cell aggregates, Myeloproliferative Neoplasms (MPN), CALR mutation, JAK2 mutation

In the published article, there was an error in the author list, and author Marlies J. W. Peeters was erroneously omitted. The corrected author list appears below.

Ana Micaela Carnaz Simões^1^, Morten Orebo Holmström^1^, Pia Aehnlich^1^, Anne Rahbech^1^, Marlies J. W. Peeters^1^, Aneta Radziwon-Balicka^1^, Carlos Zamora^2^, Tobias Wirenfeldt Klausen^1^, Vibe Skov^3^, Lasse Kjær^3^, Christina Ellervik^4-6^, Daniel El Fassi^4,7^, Silvia Vidal^2^, Hans Carl Hasselbalch^3^, Mads Hald Andersen^1,8^ and Per thor Straten*^1,8^

^1^Department of Oncology, National Center for Cancer Immune Therapy (CCIT-DK), Herlev University Hospital, Herlev, Denmark.

^2^ IIB-Sant Pau- Institut Rec. Hospital de la Santa Creu i Sant Pau, Barcelona, Spain

^3^Department of Hematology, Zealand University Hospital, Roskilde, Denmark.

^4^Department of Clinical Medicine, Faculty of Health and Medical Sciences, University of Copenhagen, Copenhagen, Denmark.

^5^Department of Laboratory Medicine, Boston Children’s Hospital, Harvard Medical School, Boston, MA, United States.

^6^Department of Data and Innovation Support, Region Zealand, Sorø, Denmark.

^7^Department of Hematology, Rigshospitalet University Hospital, Copenhagen, Denmark

^8^Department of Immunology and Microbiology, Faculty of Health and Medical Sciences, University of Copenhagen, Copenhagen, Denmark.

The authors apologize for this error and state that this does not change the scientific conclusions of the article in any way. The original article has been updated.

## Publisher’s note

All claims expressed in this article are solely those of the authors and do not necessarily represent those of their affiliated organizations, or those of the publisher, the editors and the reviewers. Any product that may be evaluated in this article, or claim that may be made by its manufacturer, is not guaranteed or endorsed by the publisher.

