# Supplementary material for: Patients With Myeloproliferative Neoplasms Harbor High Frequencies of CD8 T Cell-Platelet Aggregates Associated With T Cell Suppression
Source: Front Immunol. 2022 May 6;13:866610. doi: 10.3389/fimmu.2022.866610 (PMC9120544; doi:10.3389/fimmu.2022.866610)
Supplement: Supplementary file 1 [file DataSheet_1.pdf]

**Supplemental Material and Methods:**

## Extracellular Flow Cytometry

For extracellular flow cytometry analysis, 1 x $10^6$ cells were washed twice with phosphate-buffered saline (PBS) + 2% FBS (FACS Buffer) and stained for 30 minutes (dark, 4˚C) with 50 µl antibody mix diluted in FACS Buffer. After, cells were washed twice and acquired on an LSR II (BD Biosciences) or a NovoCyte Quanteon (ACEA Biosciences).

All flow cytometry data were analyzed with NovoExpress software (version 1.4.1) or FlowJo (version 10.8.1). For markers without distinct positive and negative population, a fluorescence-minus-one control was used to set the gate.

## Isolation of Platelets

Peripheral blood was first centrifuged at 250 g for 10 minutes (no breaks) to obtain PLT-rich plasma, which was centrifuged again (900 g, 10 minutes, no breaks). The pelleted PLT were washed and resuspended in RPMI + 10% FBS (R10). Prostaglandin I2 (Sigma) was added before each centrifugation (Peripheral blood: 0,075 µg/ml; PLT-rich plasma: 0,3 µg/ml). After one-hour rest, purity, activation, and functionality of the isolated PLT were assessed by flow cytometry. 98% of events were CD41a$^+$ (PLT marker), and 3 - 20% of PLTs expressed P-selectin (CD62P) with low levels of mean fluorescence intensity (MFI); Thrombin stimulation (1 U/ml; 10 minutes) increased PLT surface expression of P-selectin to >98% with high MFI, confirming a proper PLT functionality after isolation.

## MHC-Multimer Staining

PE- and APC-coupled empty-loadable MHC tetramers were incubated with 200 µM of HIV, CMV or FLU peptides, for 30 minutes, on ice, in the dark. Subsequently, the antigen-specific tetramers were incubated (15 minutes, 37°C, dark) with the previously prepared PBMC, before continuing to the extracellular staining procedure.

**CMV and FLU Peptides**

The CMV and FLU peptides (NLVPMVATV and GILGFVFTL) were synthesized by Schafer-N and dissolved in DMSO to a stock concentration for 10 mM. HIV peptide (ILKEPVHGV), synthesized by KJ Ross-Petersen ApS and used as a negative control, was dissolved in water to a stock concentration of 200 µM.

**Retroviral transduction of human primary T cells with gp100TCR**

For retroviral transduction, a gp100-TCR (kindly provided by the group of Reno Debets) was cloned into pMP71 (kindly provided by dr. S. Kobold, Klinikum der Ludwig-Maximilians-Universität München) via NotI and EcoRI (pMP71_gp100-alpha and pMP71_gp100-beta). A Platinum-A retroviral Packaging Cell Line (Cell Biolabs) was transfected with pMP71_gp100-alpha and pMP71_gp100-beta using Calcium Phosphate Transfection Kit (Thermo Fischer) according to manufacturer's instructions. Retroviral supernatant produced by Platinum-A cells was used for transduction of human primary T cells.

T cells were isolated from PBMC from healthy volunteers and activated by anti-CD3/CD28 beads (Dynabeads® Human T-activator CD3/CD28, Thermo Fischer Scientific). T cells were positively selected after incubation with the beads on a head-over-head tumbler (HulaMixer, Invitrogen) for 30 minutes at RT. Transduction was performed two days after T cell activation; 24-well non-tissue culture-treated plates were coated with 400 µl per well of RetroNectin reagent (Takara, Clontech) overnight at 4°C. Wells were blocked for 30 minutes with PBS+2% BSA (filtrated) and washed with PBS before 2 ml/well of virus supernatant was added (0.45 µM filtrated). The plate was centrifuged for 60 minutes at 3000 rpm at 32°C. After centrifugation, 0.5 ml of virus supernatant was left per well and 0.5 x $10^6$/ml T cells resuspended in culture media (X-vivo + 5% human serum + 100 U/ml IL-2 + 5 ng/ml IL-15) was added per well. The plate was centrifuged for 60 minutes at 2500 rpm at 32°C and incubated at 37°C, 5% $CO_2$ overnight. The next day, a second hit of transduction was performed following same procedure as described. T cells were also subjected to the same procedure, albeit without addition of plasmids (Mock transduced T cells), and used as a negative control.

After last centrifugation step, cells were incubated for six hours and then transferred to a tissue-culture treated plate. Cells were split if needed and resuspended in fresh culture media. The gp100-TCR contained the variable chain 14 (vb14), which was used as a marker for detection and purification of transduced T cells.

**Intracellular Staining**

PBMC from MPN patients ($1x10^6$ cells/ml) were cultured in low or intermediate doses of OKT-3 for five hours in the presence of Brefeldin A (BioLegend) and anti-CD107a antibody. PMA (5 ng/ml) and ionomycin (75 nM) (Sigma Aldrich) were used as a positive control. After five hours, cells were stained with cell surface antibodies as described above (Panel VI, Supplemental Table 1). After two washing steps, and according to the manufacturer instructions, cells were fixed and permeabilized overnight (Intracellular Fixation & Permeabilization Buffer Set, eBioscience). On the next day, cells were stained with intracellular antibodies as described for surface antibody staining. Lastly, cells were washed twice with Permeabilization Buffer, resuspended in FACS buffer and acquired on the NovoCyte Quanteon.

**Proliferation Assay**

PBMC from MPN patients ($1x10^6$ cells/ml) were stained with CellTrace® violet (CTV) (Thermofisher), following the manufacturer recommendations. In brief, cells were washed twice with PBS, and incubated with CTV (1:1000 dilution from stock solution) at 37°C, for 20 minutes, in the dark. After, cells were washed and resuspended in culture media. After co-culture with PLT, $2x10^6$ CTV+ PBMC were plated in a 24-well plate (Corning Costar) and stimulated with low or intermediate doses of OKT-3. Cell proliferation was evaluated after five days by flow cytometry (Panel VII, Supplemental Table 1).

**xCELLigence Real-Time Cell Analysis Assay**

The xCELLigence SP system is constituted of a E96 plate station (xCELLigence-specific 96 well plate; ACEA biosciences) kept within a standard tissue culture incubator (37°C and 5% $CO_2$). An electrode network covering the bottom of E96 plates allows this system to measure the variation of impedance over time. The xCELLigence RTCA Software Pro (ACEA Biosciences) converts the impedance values into cell index, which correlates with the strength of cell adhesion and cell number. Cell index can then be used to calculate the percentage of tumor cell lysis.

FM3 cells were plated in an E96 plate at the optimal seeding density of 7 000 cells/well (previously optimized; data not shown) and the cell index was registered every 15 minutes. After 20 hours rest to promote cancer cell adhesion and proliferation, effector cells were added: $gp100^+$ T cells alone, $gp100^+$ T cells + PLT, $gp100^+$ T cells + sPLT, and mock transduced T cells were added at different effector-to-target ratios (3:1; 1.5:1 and 0.75:1). Cell index was registered every 30 minutes for 80 hours. Data was analyzed with the immunotherapy module of the xCELLigence RTCA Software Pro as reported previously[34].

**Supplemental Table 1. Characteristics of the monoclonal antibody used in the muticolor flow cytometry panels.**

| Marker | Fluorochrome | Clone | Dilution | Company |
|---|---|---|---|---|
| **Panel I - PLT-binding to Immune Populations** | | | | |
| CD3 | PE | UCHT1 | 1:5 | BD Biosciences |
| CD4 | PE-Cy7 | SK3 | 1:20 | Biolegend |
| CD8 | FITC | RPA-T8 | 1:5 | BD Biosciences |
| CD19 | PerCP-Cy5.5 | HIB19 | 1:20 | BD Biosciences |
| CD41a | APC | HIP8 | 1:10 | BD Biosciences |
| CD56 | BV510 | HCD56 | 1:10 | Biolegend |
| CD62P | BV421 | AK-4 | 1:20 | Biolegend |
| NiR | (APC-Cy7 Channel) | | 1:500 | Invitrogen |
| **Panel II - PLT-binding in PBMC vs BMNC** | | | | |
| CD3 | AF700 | UCHT1 | 1:20 | Biolegend |
| CD4 | BV711 | OKT4 | 1:25 | Biolegend |
| CD8 | FITC | RPA-T8 | 1:5 | BD Biosciences |
| CD41a | APC | HIP8 | 1:10 | BD Biosciences |
| CD45RO | PerCP-Cy5.5 | UCHL1 | 1:10 | BD Biosciences |
| CD56 | BV510 | HCD56 | 1:10 | Biolegend |
| CD62P | BV421 | AK-4 | 1:20 | Biolegend |
| CD197 | PE-Cy7 | G043H7 | 1:10 | Biolegend |
| NiR | (APC-Cy7 Channel) | | 1:500 | Invitrogen |
| **Panel III - PLT-binding to virus-specific CD8 T cells (*Ex vivo* )** | | | | |
| CD3 | AF700 | UCHT1 | 1:20 | Biolegend |
| CD4 | PE-Cy7 | SK3 | 1:20 | Biolegend |
| CD8 | FITC | RPA-T8 | 1:5 | BD Biosciences |
| CD41a | BV421 | HIP8 | 1:20 | BD Biosciences |
| NiR | (APC-Cy7 Channel) | | 1:500 | Invitrogen |
| **Panel IV - PLT-binding to OKT-3 Stimulated CD8 T cells** | | | | |
| CD8 | FITC | RPA-T8 | 1:5 | BD Biosciences |
| CD41a | APC | HIP8 | 1:10 | BD Biosciences |
| CD45RO | PerCP-Cy5.5 | UCHL1 | 1:10 | BD Biosciences |
| CD57 | PE | HCD57 | 1:10 | Biolegend |
| CD137 | BV421 | 4B4-1 | 1:25 | Biolegend |
| CD197 | PE-Cy7 | G043H7 | 1:10 | Biolegend |
| NiR | (APC-Cy7 Channel) | | 1:500 | Invitrogen |
| **Panel V - PLT-binding to CMV-specific CD8 T cells (*In vitro* )** | | | | |
| CD3 | AF700 | UCHT1 | 1:20 | Biolegend |
| CD8 | BV605 | SK-1 | 1:20 | Biolegend |
| CD41a | FITC | HIP8 | 1:10 | eBiosciences |
| CD45RO | PerCP-Cy5.5 | UCHL1 | 1:10 | BD Biosciences |
| CD137 | BV421 | 4B4-1 | 1:25 | Biolegend |
| CD197 | PE-Cy7 | G043H7 | 1:10 | Biolegend |
| NiR | (APC-Cy7 Channel) | | 1:500 | Invitrogen |
| **Panel VI - Cytokine Release (ICS)** | | | | |
| CD3 | AF700 | UCHT1 | 1:25 | Biolegend |
| CD4 | BV711 | OKT4 | 1:10 | Biolegend |
| CD8 | BV605 | SK1 | 1:10 | Biolegend |
| CD41a | FITC | HIP8 | 1:10 | eBiosciences |
| (i) IFN-γ | BV510 | 4S.B3 | 1:25 | Biolegend |
| (i) TNF-α | PE-CF594 | Mab11 | 1:20 | BD Biosciences |
| (i) Granz B | APC | GB-11 | 1:20 | Thermo Fisher |
| CD107a | BV421 | H4A3 | 1:167 | BD Biosciences |
| NiR | (APC-Cy7 Channel) | | 1:500 | Invitrogen |

(i) = intracellular marker

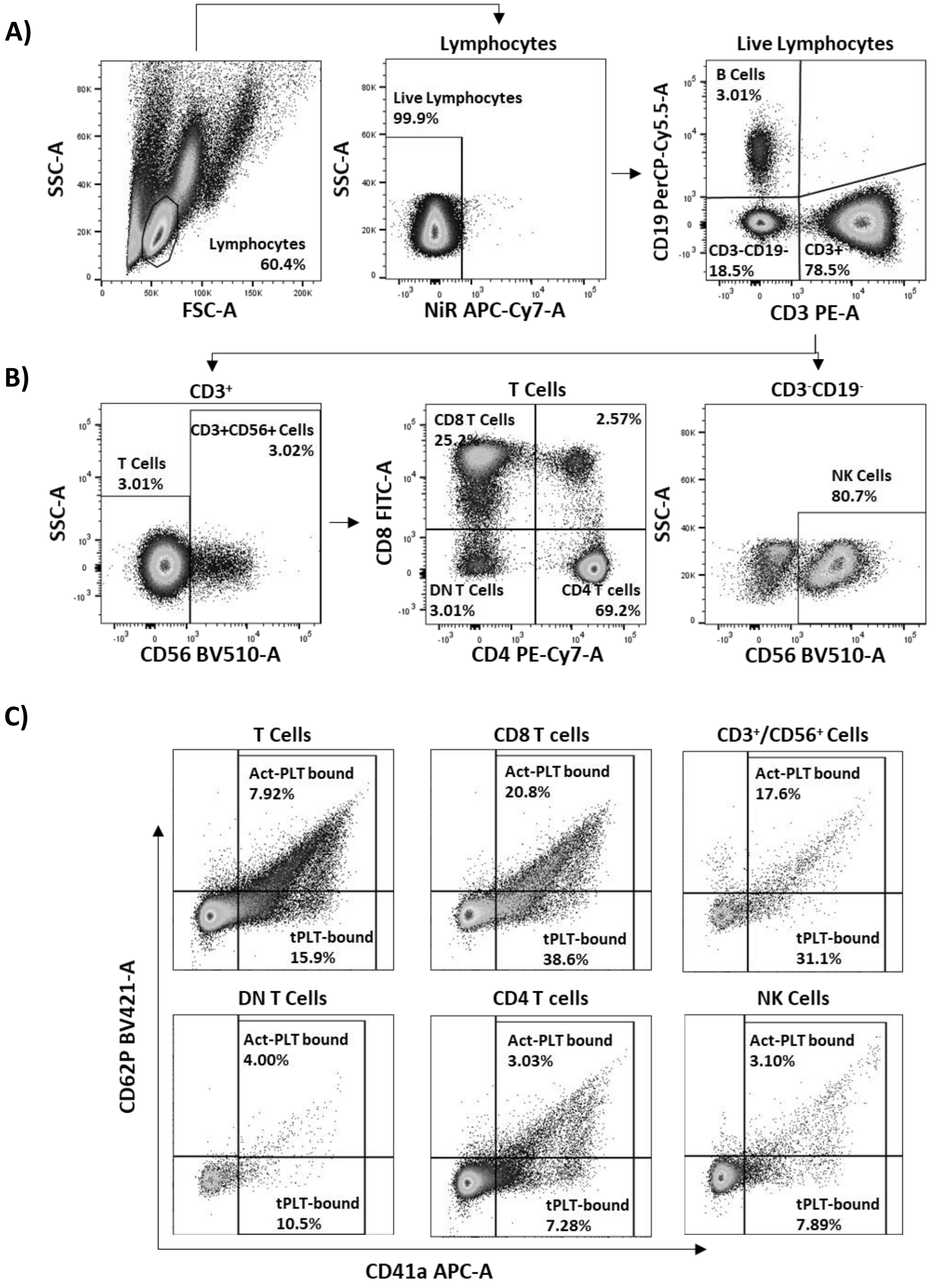


**Supplemental Figure 1. Gating Strategy for the *ex vivo* analysis of PLT-bound lymphocytes in MPN.** The gating strategy used is given for a representative MPN patient. **(A)** Live lymphocytes were gated based on their distinct size and granular properties and on the lack of Near-infra Red (NiR) dye, and further divided on their expression of CD3 and CD19. B cells were identified as $CD19^{+}/CD3^{-}$. **(B)** The $CD3^{+}/CD19^{-}$ and $CD3^{-}/CD19^{-}$populations were further separated by CD56 expression into T cells ($CD3^{+}/CD56^{-}$), $CD3^{+}/CD56^{+}$ cells and NK Cells ($CD3^{-}/CD56^{+}$). T cell subsets were identified as CD8 T cells ($CD8^{+}/CD4^{-}$) CD4 T cells ($CD8^{-}/CD4^{+}$) and DN T cells ($CD8^{-}/CD4^{-}$). **(C)** For each population mentioned above, CD41a and CD62P was used to gate the total-PLT bound populations (tPLT-bound, $CD41a^{+}$) and activated-PLT bound (act-PLT bound; $CD41a^{+}/CD62P^{+}$). Fluorescent minus one was used to set the gates on CD41a and CD62P (data not shown).

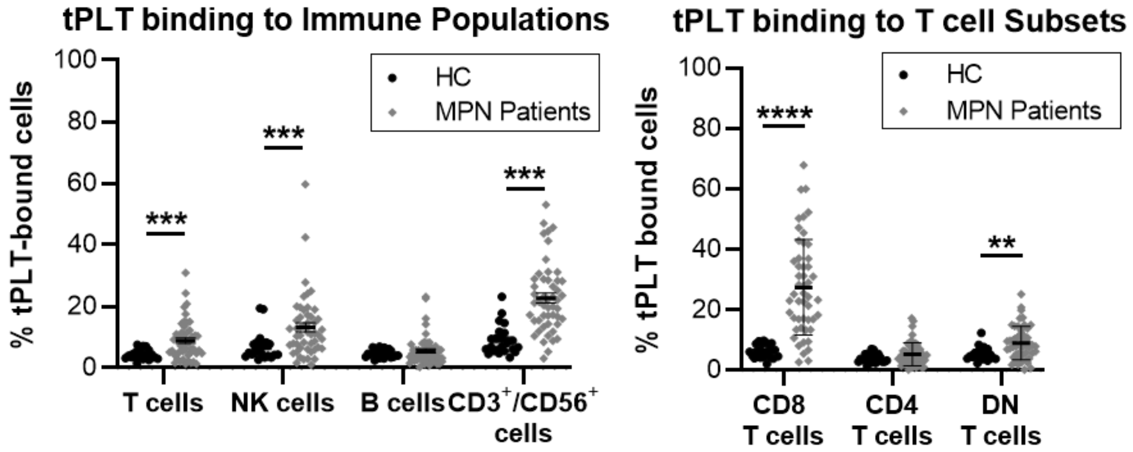


**Supplemental Figure 2. MPN patients have increased frequencies of circulating tPLT bound cells compared to HC.** Cryopreserved PBMCs from 50 patients with chronic myeloproliferative neoplasms (MPN) and 24 age-matched healthy controls (HC) were analyzed using flow cytometry (Panel I, Table S1) and the total-platelet (tPLT) binding to lymphocytes were evaluated. (Detailed gating strategy in Supplemental Figure 1) **(A)** Frequencies of tPLT binding to **(B)** the main lymphocytic populations and **(C)** the T cell subsets were compared in HC (black) and MPN patients (grey). All frequencies are shown as percentage of parent population. The horizontal lines and error whiskers represent the mean ± standard error of the mean. Unpaired T test was used to compare MPN patient and HC, Differences were considered significant when $p<0.05$, as indicated with asterisks (* $p < 0.05$, ** $p < 0.01$, *** $p < 0.001$, and **** $p < 0.0001$).

A)

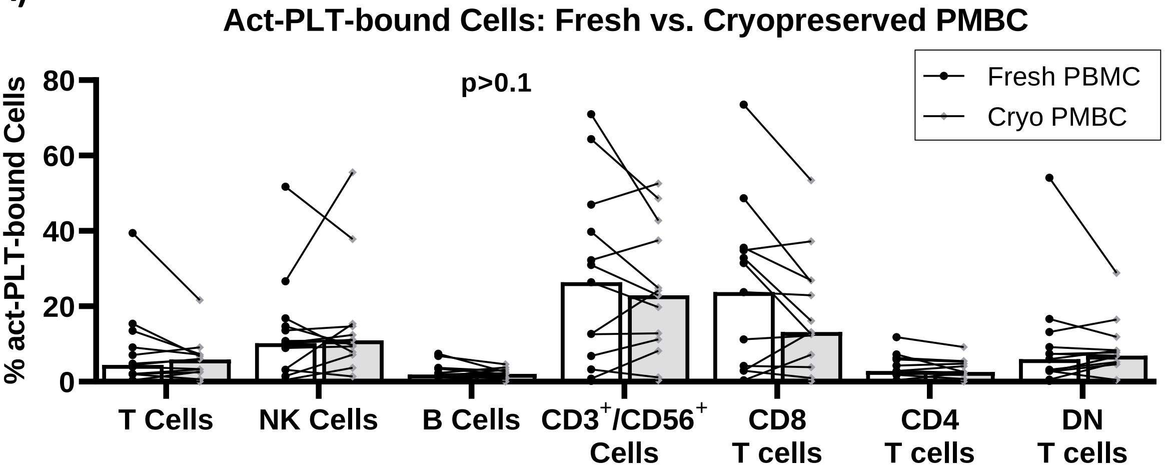


B)

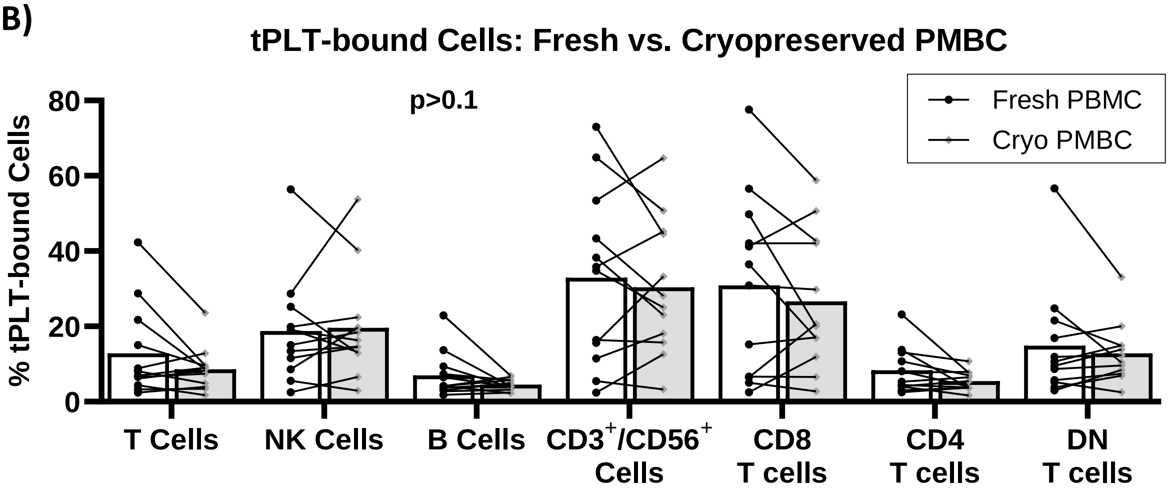


**Supplemental Figure 3. Frequency of PLT-bound immune cells is similar in freshly isolated and cryopreserved PBMC from MPN patients.** Peripheral blood mononuclear cells (PBMC) were isolated from peripheral blood of 12 MPN patients; Cells were either immediately evaluated (Fresh PBMC; black dots and empty bars) or cryopreserved for at least one month (Cryo PBMC; grey dots and grey-filled bars) before analysis. The frequencies of **(A)** activated-PLT (act-PLT)-bound lymphocytes and **(B)** total-PLT (tPLT)-bound lymphocytes were evaluated using flow cytometry (Panel I; Table S1). All frequencies are shown as percentage of parent population. The bars represent the median frequency for each population. Multiple unpaired T test was used to compared fresh and cryopreserved samples. Differences were considered significant when $p < 0.05$.

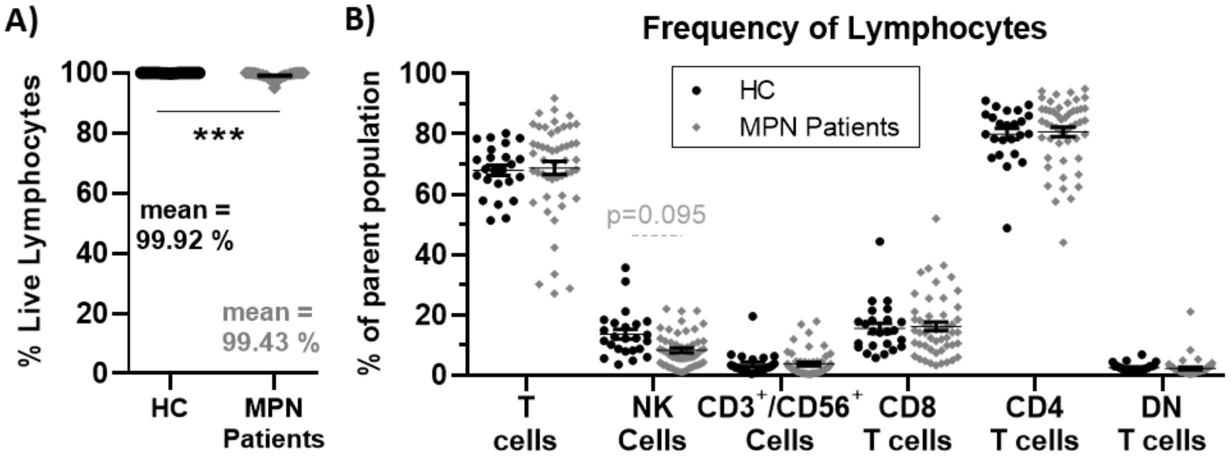


**Supplemental Figure 4. MPN and HC have comparable frequencies of lymphocytic populations.** Cryopreserved PBMCs from 50 patients with myeloproliferative neoplasms (MPN) and 24 age-matched healthy controls (HC) were analyzed using multicolor flow cytometry. The frequencies of **(A)** live cells and **(B)** T, B, natural killer (NK), $CD3^{+}/CD56^{+}$ -cells and T cell subsets were compared in MPN (grey) and HC (black). All frequencies are shown as percentage of parent population and the horizontal lines and error whiskers represent the mean ± standard error of the mean. Multiple unpaired T test was used to compare MPN patient and HC, and differences were considered significant when p<0.05, as indicated with asterisks (* p<0.05, ** p<0.01, *** p<0.001, and **** p<0.0001). Doted lines were used when 0.1< p value <0.05 to identify possible trends among populations.

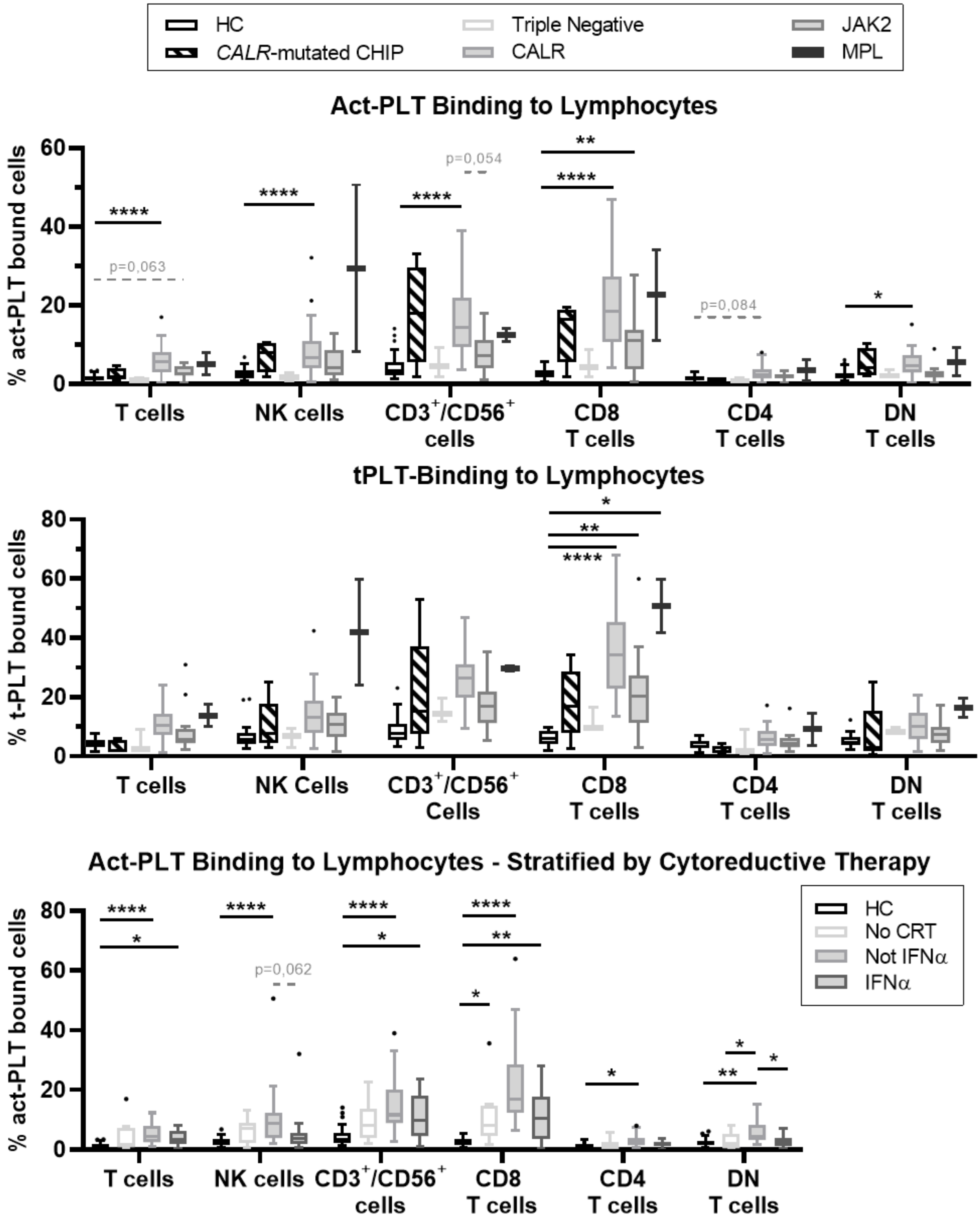


**Supplemental Figure 5. Patients harboring *CALR* mutations and receiving CRT other than IFNα have high frequencies of PLT-lymphocyte aggregates.** Frequencies of PLT-bound lymphocytes were compared between age matched healthy controls (HC, n=24, grey bars) and MPN patients. The patient population was stratified by mutation – triple negative (n=3), *CALR* (n=25), *JAK2* (n=16) and *MPL* (n=2) and the frequencies of **(A)** act-PLT bound and **(B)** tPLT bound lymphocytes was compared; and **(B)** cytoreductive therapy (CRT) – No CRT (n=8), CRT that is not interferon- alpha (Not IFNα; n=22) and IFNα (n=16). *CALR*-mutated CHIP (n=4) were included and represent asymptomatic individuals with *CALR* mutations. Frequencies are shown as percentage of parent population and represented by Tukey's box and whiskers. Kruskal-wallis test was used to compare the groups with $n \geq 8$ and differences were considered significant when $p < 0.05$ (* $p < 0.05$, ** $p < 0.01$, *** $p < 0.001$, and **** $p < 0.0001$). Doted lines were used when $0.1 < p$ value $< 0.05$.

**Supplemental Table 2. Linear regression analysis between the frequency of PLT-binding and the clinical characteristics.** 41 MPN patients with *CALR* or *JAK2* mutations were used for regression analysis. The correlation between clinical characteristics (independent variables) and the frequency of total- (t) or activated (act-) PLT bound cells (dependent variables) was analyzed, using single linear regressions (Fig 2A and Fig S5, respectively). Here we show the detailed linear regression analysis for the relevant linear regressions ($R^2$ > 0.10 and p-value < 0.05). The regression line and the goodness of fit ($R^2$ value and associated p-value) for each linear regression model is shown. Furthermore, when dummy variables were used (binominal independent variables), description of the dummy code is provided. This way the variance in PLT-binding due to each independent variable can be calculated. Of note, to ensure that the data followed a normal distribution during the linear regression analysis, the dependent variables were log transformed. This means that when calculating the variance in PLT-binding the results will need to be transformed back to percentage.

| Dependent Variable | Independent Variable(s) | Regression Line | Goodness of Fit | | Dummy variables |
|---|---|---|---|---|---|
| | | | $R^2$ | p-value | |
| Log (% aPLT-bound CD8 T cells) | PLT Count | Y = 0.0009259 X + 0.7699 | 0.1487 | 0.0128 | |
| Log (% aPLT-bound NK cells) | PLT Count | Y = 0.0009919 X + 0.3819 | 0.1911 | 0.0043 | |
| Log (% aPLT-bound T cells) | PLT Count | Y = 0.0008829 X + 0.2562 | 0.1507 | 0.0121 | |
| Log (% aPLT-bound CD4 T cells) | PLT Count | Y = 0.001005 X - 0.08818 | 0.1614 | 0.0092 | |
| Log (% aPLT-bound CD8 T cells) | Mutation | Y = - 0.348 X + 1.223 | 0.1916 | 0.0042 | X(CALR) = 0; X(JAK2) = 1 |
| Log (% aPLT-bound CD3/CD56 T cells) | Mutation | Y = - 0.3274 X + 1.147 | 0.2419 | 0.0011 | X(CALR) = 0; X(JAK2) = 1 |
| Log (% tPLT-bound CD8 T cells) | PLT Count & Mutation | Y = 0.0008051 $X_1$ - 0.3141 $X_2$ + 0.9339 | 0.211 | 0.0025 | $X_1$ = PLT count (continuous)<br>$X_2$(CALR) = 0; $X_2$(JAK2) = 1 |
| Log (% tPLT-bound CD8 T cells) | PLT Count | Y = 0.0008166 X + 1.092 | 0.211 | 0.0025 | |
| Log (% tPLT-bound NK cells) | PLT Count | Y = 0.001007 X + 0.6538 | 0.2596 | 0.0007 | |
| Log (% tPLT-bound T cells) | PLT Count | Y = 0.0008194 X + 0.5913 | 0.1764 | 0.0063 | |
| Log (% tPLT-bound CD4 T cells) | PLT Count | Y = 0.0009145 X + 0.3066 | 0.1654 | 0.0083 | |
| Log (% tPLT-bound CD8 T cells) | Mutation | Y = - 0.2194 X + 1.458 | 0.139 | 0.0164 | X(CALR) = 0; X(JAK2) = 1 |

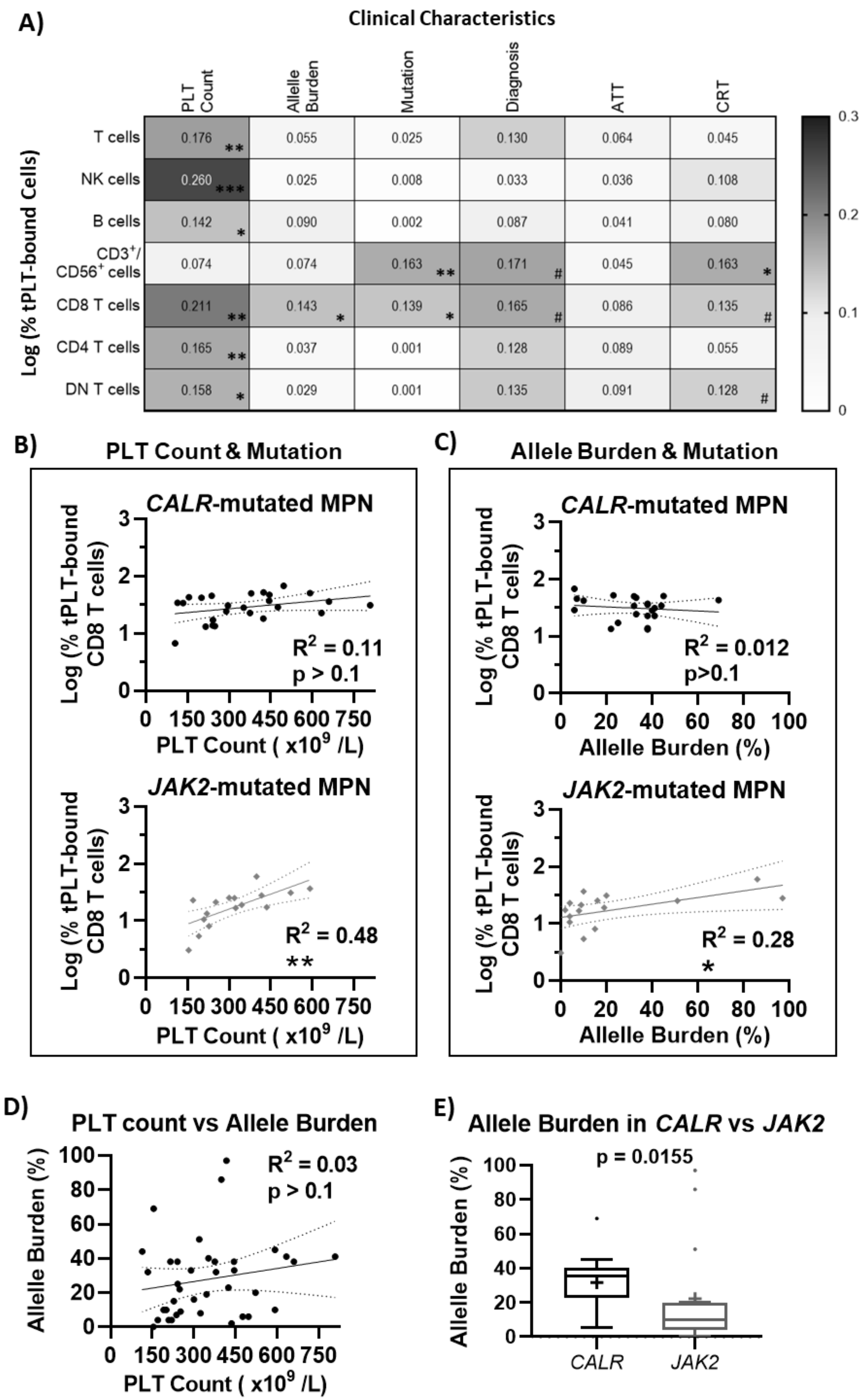
A)
Clinical Characteristics
PLT Count
Allele Burden
Mutation
Diagnosis
ATT
CRT
Log (% tPLT-bound Cells)
T cells 0.176 ** 0.055 0.025 0.130 0.064 0.045
NK cells 0.260 *** 0.025 0.008 0.033 0.036 0.108
B cells 0.142 * 0.090 0.002 0.087 0.041 0.080
CD3+/CD56+ cells 0.074 0.074 0.163 ** 0.171 # 0.045 0.163 *
CD8 T cells 0.211 ** 0.143 * 0.139 * 0.165 # 0.086 0.135 #
CD4 T cells 0.165 ** 0.037 0.001 0.128 0.089 0.055
DN T cells 0.158 * 0.029 0.001 0.135 0.091 0.128 #
0.3
0.2
0.1
0
B)
PLT Count & Mutation
CALR-mutated MPN
R2 = 0.11
p > 0.1
JAK2-mutated MPN
R2 = 0.48
**
Log (% tPLT-bound CD8 T cells)
PLT Count ( x109 /L)
C)
Allele Burden & Mutation
CALR-mutated MPN
R2 = 0.012
p>0.1
JAK2-mutated MPN
R2 = 0.28
*
Log (% tPLT-bound CD8 T cells)
Allele Burden (%)
D)
PLT count vs Allele Burden
R2 = 0.03
p > 0.1
Allele Burden (%)
PLT Count ( x109 /L)
E)
Allele Burden in CALR vs JAK2
p = 0.0155
Allele Burden (%)
CALR
JAK2

**Supplemental Figure 6. (previous page) PLT Count and allele burden correlate with the frequency of tPLT-bound-CD8 T cells in *JAK2*- but not *CALR*-mutated MPN patients.** 41 MPN patients with *CALR* or *JAK2* mutations were used for regression analysis. **(A)** The correlation between clinical characteristics (column factors) and the frequency of total-PLT (tPLT) bound cells (row factors) was analyzed, using single linear regressions. The goodness of fit ($R^2$ value) for each linear regression model is shown as a heatmap ($0 < R^2 < 0.3$). The models were considered relevant when $R^2 > 0.10$ and p-value < 0.05; The correlation between **(B)** PLT count and **(C)** mutant allele burden and the frequency of tPLT-bound CD8 T cells was analyzed by linear regression and independently for *CALR*- and *JAK2*- mutated patients. Doted lines represent the 95% confidence bands of the best-fit line and for each line the $R^2$ was calculated. **(D)** To exclude the effect of confounding factors, the correlation between PLT count and Allele burden was also evaluated with linear regression. **(E)** The frequency of allele burden is shown for *CALR*- and *JAK2*-mutated MPN patients. Unpaired T test was used to compare the two populations. Differences between groups were considered significant when $p < 0.05$. Asterisks represent * $p < 0.05$, ** $p < 0.01$, *** $p < 0.001$, and **** $p < 0.0001$. PLT = Platelets; MPN = Chronic Myeloproliferative Neoplasms; AAT = Anti-thrombotic Therapy; CRT = Cytoreductive Therapy.

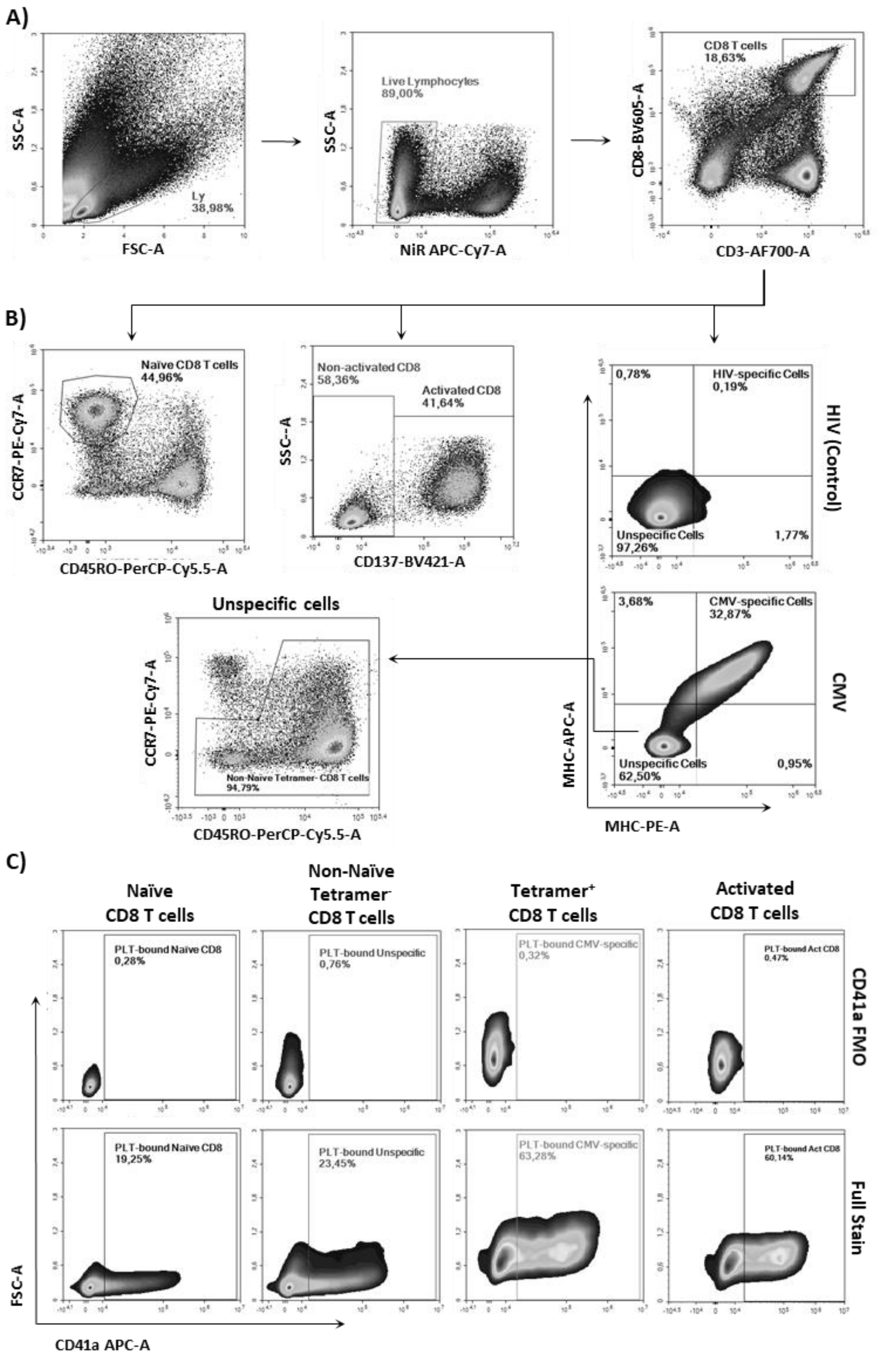


**Supplemental Figure 7. Gating Strategy for the analysis of *in vitro* PLT-binding to virus-specific CD8 T cells.** PBMC from healthy volunteers were stimulated twice with a CMV peptide and later co-cultured with PLT (PBMC:PLT ratio was 1:100). PLT-binding was evaluated using flow cytometry. The gating strategy used is given for a representative sample. **(A)** Live Lymphocytes were gated based on their distinct size and granular properties and lack of Near-infra Red (NiR) dye. CD8 T cells were identified based on their co-expression of CD3 and CD8. **(B)** Antigen-specific CD8 T cells were identified by tetramer staining. HIV-tetramers were used as a negative control for CMV-specific CD8 T cells (Tetramer$^{+}$ CD8 T cells). Naïve CD8 T cells (CCR7$^{+}$/CD45RO$^{-}$), non-activated (CD137$^{-}$) and activated (CD137$^{+}$) CD8 T cells were also gated from the total CD8 T cells. Non-Naïve Tetramer$^{-}$ CD8 T cells were gated as CCR7$^{+}$/CD45RO$^{\pm}$ of total unspecific cells. **(C)** For each of these populations, CD41a was used to gate PLT-bound populations (CD41a$^{+}$). Fluorescent minus one (FMO) was used to set the gates on CD41a.
